# Redundancy of human ATG4 protease isoforms in autophagy and LC3/GABARAP processing revealed in cells

**DOI:** 10.1080/15548627.2019.1569925

**Published:** 2019-02-01

**Authors:** Alexander Agrotis, Niccolo Pengo, Jemima J. Burden, Robin Ketteler

**Affiliations:** MRC Laboratory for Molecular Cell Biology, University College London, London, UK

**Keywords:** Atg8, CLEM, CRISPR, delipidation, GABARAPL2, knockout

## Abstract

Macroautophagy/autophagy is a cellular degradation pathway that delivers cytoplasmic material to lysosomes via double-membrane organelles called autophagosomes. Lipidation of ubiquitin-like LC3/GABARAP proteins on the autophagosome membrane is important for autophagy. The cysteine protease ATG4 executes 2 LC3/GABARAP processing events: priming of newly synthesized pro-LC3/GABARAP to enable subsequent lipidation, and delipidation/deconjugation of lipidated LC3/GABARAP (the exact purpose of which is unclear in mammals). Four ATG4 isoforms (ATG4A to ATG4D) exist in mammals; however, the functional redundancy of these proteins in cells is poorly understood. Here we show that human HAP1 and HeLa cells lacking ATG4B exhibit a severe but incomplete defect in LC3/GABARAP processing and autophagy. By further genetic depletion of ATG4 isoforms using CRISPR-Cas9 and siRNA we uncover that ATG4A, ATG4C and ATGD all contribute to residual priming activity, which is sufficient to enable lipidation of endogenous GABARAPL1 on autophagic structures. We also demonstrate that expressing high levels of pre-primed LC3B in ATG4-deficient cells can rescue a defect in autophagic degradation of the cargo receptor SQSTM1/p62, suggesting that delipidation by human ATG4 is not essential for autophagosome formation and fusion with lysosomes. Overall, our study provides a comprehensive characterization of ATG4 isoform function during autophagy in human cells.

**Abbreviations:** Atg: autophagy-related; baf A1: bafilomycin A_1;_ CASP3: caspase 3; CLEM: correlative light and electron microscopy; CMV: cytomegalovirus; CRISPR: clustered regularly interspaced short palindromic repeats; DKO: double knockout; EGFP: enhanced green fluorescent protein; GABARAP: GABA type A receptor-associated protein; GABARAPL1: GABA type A receptor-associated protein like 1; GABARAPL2: GABA type A receptor-associated protein like 2; GFP: green fluorescent protein; HB: homogenization buffer; KO: knockout; LAMP1: lysosomal associated membrane protein 1; LIR: LC3 interacting region; MAP1LC3/LC3: microtubule-associated protein 1 light chain 3; MFN2: mitofusin 2; N.A.: numerical aperture; NEM: N-ethylmaleimide; PDHA1: pyruvate dehydrogenase E1 alpha 1 subunit; PLD: phospholipase D; PE: phosphatidylethanolamine; RLUC: Renilla luciferase; SQSTM1: sequestosome 1; TEM: transmission electron microscopy; TKO: triple knockout; ULK1: unc-51 like autophagy activating kinase 1; VCL: vinculin; WT: wild-type

## Introduction

Macroautophagy (hereafter autophagy) is a conserved process in eukaryotic cells that delivers cytoplasmic material including protein aggregates and damaged organelles to lysosomes for degradation. It involves the formation of a cup-shaped membrane (phagophore) that expands to surround the cargo to be degraded. Once fully closed, the resulting double-membrane organelle (autophagosome) undergoes fusion with endosomes and lysosomes. This eventually leads to degradation of inner autophagosome membrane and its contents, allowing recycling of macromolecules within. Autophagy serves to mobilize nutrients during starvation, as well as being important for pathogen defense, cellular remodeling and proteostasis [1,2].

The genes that regulate autophagy, termed autophagy-related (*Atg*) genes, were first identified in yeast [3,4], leading to functional studies into the molecular mechanisms of Atg proteins. These discoveries revealed that most Atg proteins cluster into 1 of 5 ‘core machinery’ components that act in largely consecutive steps during autophagosome formation: the ULK protein kinase complex, BECN1/beclin1-PIK3C3/Vps34 lipid kinase complex, the Atg9 trafficking system, and the Atg12–Atg5 and Atg8–PE ubiquitin-like conjugation systems. In the latter, the small ubiquitin-like protein Atg8 (LC3/GABARAP in mammals) is covalently conjugated to phospholipids such as phosphatidylethanolamine (PE) on the phagophore membrane in a reaction mechanism similar to ubiquitin conjugation [5]. This lipidation reaction is catalysed by E1-like activating enzyme Atg7, E2-like conjugating enzyme Atg3 and enhanced by the E3-like Atg12–Atg5 conjugate formed in the preceding reaction [6]. Lipidation of Atg8 is essential for autophagy because it contributes to autophagosome biogenesis [7], as well as recruitment of specific autophagy cargo via cargo receptors that bind directly to Atg8 such as the ubiquitin-binding protein SQSTM1/p62 [8]. The cysteine protease Atg4 is thought to regulate Atg8 lipidation through 2 processing steps. First, newly synthesized Atg8 undergoes priming/activation by proteolytic cleavage of its C-terminus to expose a specific glycine residue that is subsequently targeted to PE during lipidation [9]. Second, Atg4 can hydrolyze the amide linkage between Atg8 and PE in a delipidation/deconjugation step that recycles free Atg8. In yeast, this delipidation reaction is proposed to be important for full autophagic flux and autophagosome formation, while additionally preventing the mislocalization of lipidated LC3 to other membranes in the cell [10–12]. Whether the role of delipidation is conserved in mammalian cells remains to be determined.

Mammalian genomes encode multiple isoforms of Atg4 and Atg8. The Atg8 proteins are divided into two subfamilies: LC3 (MAP1LC3A, B, B2 and C) and GABARAP (GABARAP, GABARAPL1 and GABARAPL2). Recently, LC3/GABARAP has been implicated in inner autophagosome membrane degradation [13], and in a separate study these proteins were implicated in autophagosome-lysosome fusion [14]. This is in contrast to an earlier study that proposed the different subfamilies to play essential and distinct roles in phagophore elongation and closure [15]. Despite growing interest in LC3/GABARAP isoform function, it remains unclear which of the mammalian ATG4 isoforms (ATG4A, B, C and D) are responsible for LC3/GABARAP processing and autophagy in cells. ATG4B is considered to be the main isoform since studies *in vitro* show that it has the most activity and broadest specificity towards cleaving different isoforms of synthetic tagged LC3/GABARAP constructs [16]. ATG4A has been shown to be capable of processing GABARAP subfamily isoforms [17], but with a reduced activity compared to ATG4B [16]. In contrast, ATG4C and ATG4D exhibit almost no *in vitro* activity [16], but the activity of ATG4D in cells might be enhanced through N-terminal cleavage mediated by the apoptosis-regulating protease CASP3/caspase-3 [18]. Although mice lacking ATG4B show reduced processing of murine LC3/GABARAP orthologs, they survive to adulthood with a balance disorder suggesting they suffer from an impairment rather than complete defect in autophagy [19]. This is in contrast to ATG3-deficient mice which completely lack LC3/GABARAP lipidation and die from starvation shortly after birth [20]. However it is not known which of the other ATG4 isoforms could contribute to LC3/GABARAP processing in the absence of ATG4B.

In this study, we performed a detailed characterization of human cells lacking ATG4B to determine its role in autophagy. We show that loss of ATG4B causes severe defects in autophagy and LC3/GABARAP processing, however the remaining ATG4 activity is sufficient for residual lipidation and autophagosome localization of GABARAP subfamily isoforms. By further depletion of ATG4 isoforms, we discover that ATG4A, ATG4C and ATGD all contribute to the remaining processing activity and thus show overlapping redundancy in cells. We also investigate roles of ATG4-mediated delipidation by rescuing ATG4-deficient cells with high-level expression of pre-primed LC3/GABARAP, uncovering that ATG4-mediated delipidation is not essential for autophagosome formation or lysosome fusion.

## Results

### ATG4B is required for LC3B lipidation but not GABARAPL1 and GABARAPL2 lipidation

**Figure 1.**
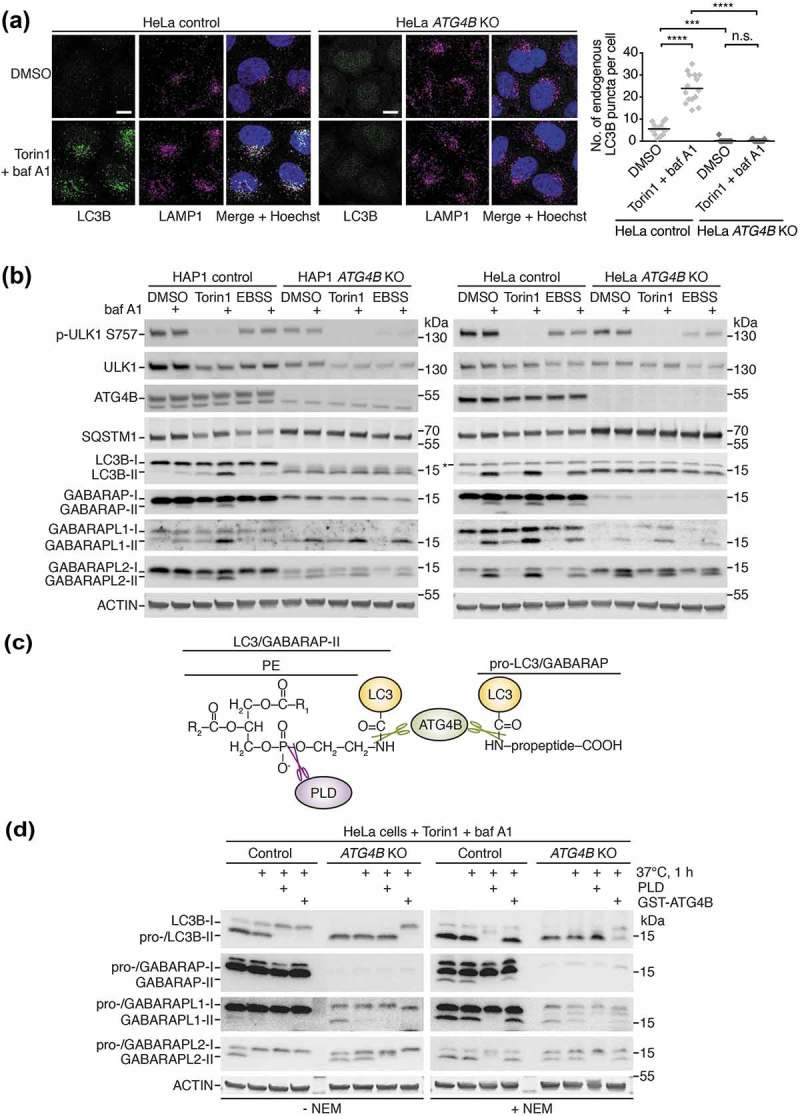
ATG4B is required for LC3B lipidation but not GABARAP isoform lipidation. (**a**) Localization of endogenous LC3B and LAMP1 in HeLa control and *ATG4B* KO cells treated for 3 h with DMSO or 250 nM Torin1 + 10 nM bafilomycin A_1_ (baf A1) revealed by immunocytochemistry. Scale bar: 10 µm. Quantification of LC3B puncta per cell shown on right hand side. n = 15 randomly-selected cells per condition. **** P ≤ 0.0001, *** P ≤ 0.001, n.s. (not significant) P > 0.05 (Sidak’s multiple comparison test). (**b**) Western blotting of lysates from HAP1 and HeLa control and *ATG4B* KO cells treated for 3 h with either 250 nM Torin1 or EBSS in the presence or absence of 10 nM baf A1. Lysis was performed in the presence of 20 mM NEM (N-ethylmaleimide) since this was required to stabilize lipidated GABARAP and GABARAPL1 (see also **Figure S1E**). Background band detected by LC3B antibody is indicated with an asterisk (see also **Figure S1B**). (**c**) Diagram showing bonds present at the C terminus of pro- and lipidated (PE-conjugated) LC3/GABARAP that can be cleaved by ATG4B or PLD *in vitro* (not to scale). This principle provides the basis for distinguishing between the pro-LC3/GABARAP and lipidated LC3/GABARAP by western blot. (**d**) PLD bandshift assay performed on HeLa control or *ATG4B* KO cells treated for 3 h with 250 nM Torin1 + 10 nM baf A1 and lysed in PLD assay buffer with or without 20 mM NEM. Lysates were subject to *in vitro* treatment with purified PLD or GST-ATG4B prior to western blotting.

In order to dissect the function of ATG4B in autophagy, we obtained human HAP1 cells lacking ATG4B. We previously reported that these cells exhibit a complete absence of endogenous LC3B puncta as detected by immunofluorescence, in contrast to the same cells rescued with ectopic expression of wild-type ATG4B (but not catalytic-inactive C74S mutant) that showed a strong accumulation of LC3B puncta when co-treated with the autophagy inducer Torin1 and lysosome inhibitor bafilomycin A_1_ (baf A1) [21]. This observation prompted us to determine the mechanism behind loss of LC3B puncta in ATG4B-deficient cells, and to explore whether this phenotype was reproducible in a more widely characterized human autophagy cell model. To this end, we generated HeLa cells lacking ATG4B using CRISPR-Cas9, with complete loss of ATG4B protein confirmed by western blotting (**Figure S1A**). Indeed, *ATG4B* KO HeLa cells showed an absence of LC3B puncta both basally and in response to treatment with Torin1 and baf A1 (Figure 1(a)), in contrast to wild-type (control) HeLa cells, which exhibited bright puncta of endogenous LC3B that accumulated and colocalized with the lysosome marker LAMP1 in response to treatment.

To investigate the overall effect of ATG4B loss on the processing of endogenous LC3B, we treated *ATG4B* KO and wild-type counterpart (control) HAP1 and HeLa cells with starvation medium (EBSS) or Torin1 in combination with baf A1 to assess autophagic flux, and analyzed cell lysates by western blotting. As shown in Figure 1(b), treatment of wild-type cells with baf A1 resulted in expected increased levels of the lower band of LC3B corresponding to the lipidated form, LC3B-II (LC3B–PE). There was a further increase in these lipidation levels upon co-treatment with Torin1, consistent with an increase in autophagic flux. Interestingly, starvation did not appear to stimulate further lipidation of LC3B, possibly since it was less efficient than Torin1 at causing de-phosphorylation of the upstream kinase ULK1 at its inhibitory phosphorylation site (Ser757) [22] (Figure 1(b)). Strikingly, in *ATG4B* KO cells we only observed a single specific band of LC3B that showed a similar migration to LC3B-II (Figure 1(b)). Note that the band marked by an asterisk at the height of LC3B-I in HeLa *ATG4B* KO cells was not detected using other LC3B antibodies and thus was deemed to be non-specific (see **Figure S1B**). The specific band seen in *ATG4B* KO cells did not accumulate further in response to Torin1 or baf A1 treatment, despite Torin1 treatment efficiently leading to dephosphorylation of ULK1. Cells lacking ATG4B also showed an accumulation of SQSTM1 and a reduced clearance of this protein upon Torin1 treatment, suggestive of an autophagy defect (Figure 1(b)). As human pro-LC3B was recently described to have a similar migration rate to lipidated LC3B by SDS-PAGE [23], we suspected that the single lower band of LC3B might correspond to pro-LC3B and thus potentially explain the impairment in autophagy.

In order to distinguish between endogenous pro-LC3B and LC3B-II, we devised an assay in which lysates of cells treated with Torin1 and baf A1 were subject to treatment with recombinant ATG4B or PLD (phospholipase D) prior to western blot analysis. Because PLD cleaves a bond that is only present in lipidated forms of LC3/GABARAP [24], a migration shift upon PLD treatment is indicative of the presence of the lipidated species (Figure 1(c)). As a control, recombinant ATG4B can cleave both pro- and lipidated LC3/GABARAP revealing any potential migration shift from both of these forms (Figure 1(c)). The predominant LC3B band in HeLa *ATG4B* KO cells was completely resistant to PLD treatment but underwent a migratory shift with ATG4B treatment, in contrast to the corresponding band that was sensitive to both PLD and ATG4B in wild-type cells (Figure 1(d)). This demonstrates that *ATG4B* KO cells accumulate pro-LC3B rather than LC3B-II, an observation that was also confirmed in HAP1 cells treated with baf A1 (**Figure S1C**). Therefore, ATG4B is essential for the priming of pro-LC3B and its subsequent lipidation, explaining the absence of LC3B puncta in *ATG4B* KO cells.

We could also determine the effect of ATG4B loss on GABARAP subfamily isoforms in the same set of experiments, using specific antibodies to detect endogenous GABARAP, GABARAPL1 and GABARAPL2. We observed that total levels of free GABARAP and GABARAPL1 were dramatically reduced in *ATG4B* KO cells compared to wild-type cells (Figure 1(b)), consistent with a recently discovered role of the C-terminal LC3 interacting region (LIR) of murine ATG4B in stabilizing the non-lipidated forms of these proteins and reducing their proteasomal degradation [25]. Our data suggest this function is conserved in human cells, since proteasome inhibition by treatment with the compound MG132 could also raise GABARAP protein levels in *ATG4B* KO HeLa cells (**Figure S1D**). In respect to lipidation, we could observe an accumulation of lower bands of all GABARAP isoforms in wild-type (control) HAP1 and HeLa cells in response to Torin1 and baf A1 treatment (Figure 1(b)), consistent with all of these proteins being involved in autophagy. Despite their levels generally being reduced in *ATG4B* KO cells, the lower bands of GABARAPL1 and GABARAPL2 still accumulated in response to treatment. To determine whether these lower bands corresponded to the lipidated forms of GABARAP proteins, we also detected endogenous GABARAP isoforms in PLD assay samples (Figure 1(d)). Indeed, the lower bands of GABARAPL1 and GABARAPL2 were sensitive to PLD treatment in both control and *ATG4B* KO HeLa cells, confirming that they corresponded to the lipidated forms of the proteins. This result could only be determined when the assay buffer contained the irreversible ATG4 inhibitor N-ethylmaleimide (NEM) [9] otherwise the lower band was absent or reduced by heat treatment alone presumably due to processing by endogenous ATG4 activity in the lysate. In fact, it proved essential to include NEM in the lysis buffer for all western blot experiments assessing GABARAP and GABARAPL1 lipidation, since the lipidated forms of these proteins were particularly unstable in wild-type cell lysates lacking NEM (**Figure S1E**).

Collectively, our data show that human cells lacking ATG4B fail to form lipidated LC3B due to a defect in the initial priming/activation of LC3B, and thus accumulate unprocessed pro-LC3B. However the presence of lipidated GABARAPL1 and GABARAPL2 in *ATG4B* KO cells suggests that these isoforms can be processed independently of ATG4B.

### Loss of ATG4B leads to a severe but incomplete defect in LC3/GABARAP priming

**Figure 2.**
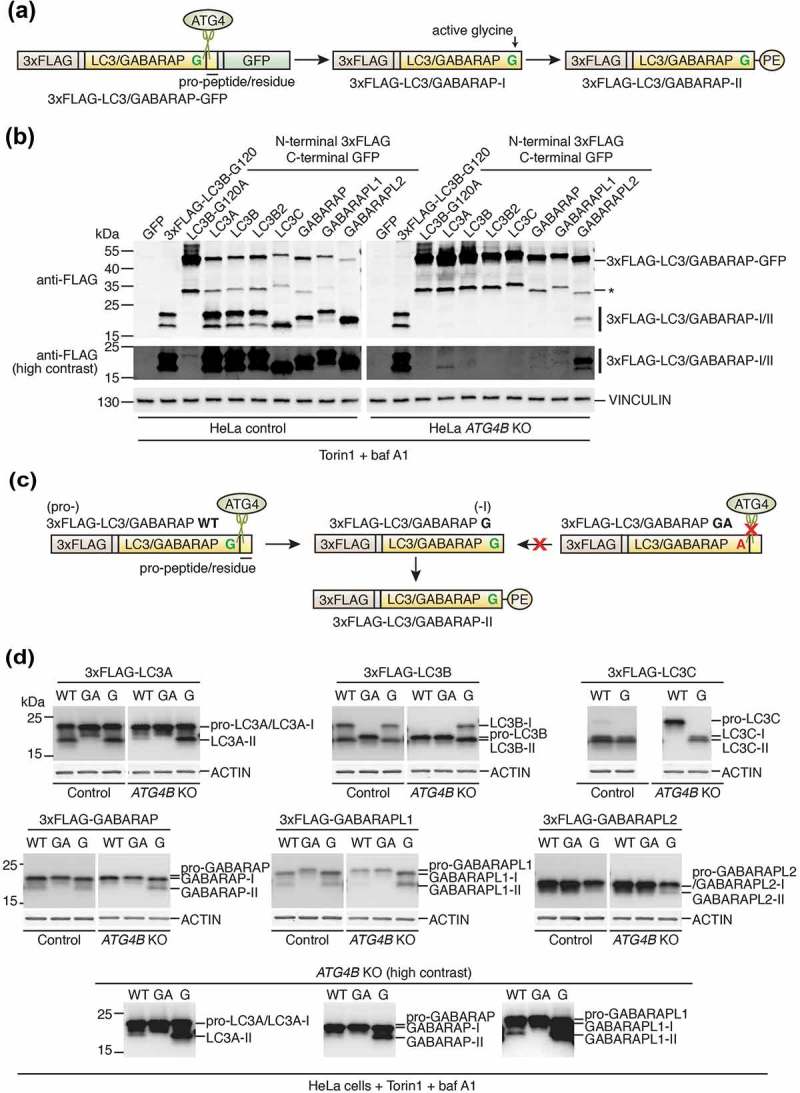
Loss of ATG4B leads to a severe but incomplete defect in LC3/GABARAP priming. **(a)** Schematic of N-terminal 3xFLAG C-terminal GFP double-tagged LC3/GABARAP constructs used in Figure 2(b) (not to scale). Cleavage site targeted by ATG4 in cells is indicated, resulting in formation of primed/activated 3xFLAG-LC3/GABARAP-I. The primed form can then undergo lipidation in cells to form 3xFLAG-LC3/GABARAP-II. (**b**) Western blotting of lysates from HeLa control and *ATG4B* KO cells transiently transfected with the indicated 3xFLAG-LC3/GABARAP-GFP, positive (3xFLAG-LC3B-G120) and negative (3xFLAG-LC3B-G120A-GFP) control constructs. At 24 h post-transfection, cells were treated for 3 h with 250 nM Torin1 + 10 nM baf A1 prior to lysis. Specific cleaved and lipidated products arising from ATG4 activity are indicated as 3xFLAG-LC3/GABARAP-I/II. The band marked with an asterisk corresponds to a non-specific cleavage product. (**c**) Schematic of N-terminal 3xFLAG-tagged LC3/GABARAP constructs used in Figure 2(d) (not to scale). Cleavage site in wild-type (WT) constructs targeted by ATG4 in cells is indicated, resulting in formation of primed 3xFLAG-LC3/GABARAP-I. To bypass cleavage, 3xFLAG-LC3/GABARAP G constructs were expressed that lack the C-terminal pro-peptide/residue. The primed form can then undergo lipidation in cells to form 3xFLAG-LC3/GABARAP-II. Mutation of the active glycine residue to alanine in 3xFLAG-LC3/GABARAP GA blocks priming, therefore this construct corresponds to the pro- form of the protein and cannot undergo conversion to 3xFLAG-LC3/GABARAP-I or further conversion to 3xFLAG-LC3/GABARAP-II. (**d**) Western blotting of lysates from HeLa control and *ATG4B* KO cells transiently transfected with indicated 3xFLAG-LC3/GABARAP wild-type (WT), primed (G) and uncleavable (GA) constructs. At 24 h post-transfection, cells were treated for 3 h with 250 nM Torin1 + 10 nM baf A1 prior to lysis. Anti-FLAG antibody was used to detect the expressed constructs. Presented as a comparison between control and *ATG4B* KO cells for each individual LC3/GABARAP, see also Figure S2 for original blots.

To determine whether differences in LC3/GABARAP lipidation seen between *ATG4B* KO and wild-type cells could be explained by impaired priming activity, we generated constructs encoding all LC3/GABARAP isoforms tagged at the N terminus with the 3xFLAG epitope and at the C terminus with GFP (Figure 2(a)). These constructs undergo a large molecular weight shift that is easily detectable by western blotting when cleaved at the C terminus by ATG4 in cells. This approach was necessary for comparing the effect of ATG4B on processing of each individual LC3/GABARAP isoform, since some commercial antibodies cannot distinguish between endogenous isoforms that are very similar in peptide sequence. As a positive control we expressed 3xFLAG-LC3B-G120 that lacks a C-terminal pro-peptide and therefore corresponds to primed LC3B, and as a negative control we expressed 3xFLAG-LC3B-G120A-GFP that cannot be primed due to mutation of its active glycine residue to alanine. As seen in Figure 2(b), all LC3/GABARAP isoforms were processed in an ATG4B-dependent manner since efficient priming occurred in wild-type cells but was abolished or reduced in cells lacking ATG4B. These constructs appeared to undergo non-specific cleavage within the GFP sequence (band marked with asterisk) but nonetheless ATG4-dependent cleavage could clearly be distinguished by the presence of bands in the 15–25 kDa range (Figure 2(b)). Of note, it was possible to detect slight processing of GABARAPL2 and an even smaller amount of LC3A processing in cells lacking ATG4B. Very small amounts of processed GABARAP and GABARAPL1 could also be observed in *ATG4B* KO cells, but higher contrast western blot images were required to detect this processing (Figure 2(b)), which is possibly explained by the relative instability of these isoforms due to enhanced proteasomal degradation when ATG4B is missing [25].

To characterize the relative SDS-PAGE migration of each form (pro vs. -I vs. -II) of LC3/GABARAP isoform, and as an alternative approach to investigate LC3/GABARAP processing by ATG4B without relying on C-terminal tags, we also generated full-length LC3/GABARAP constructs tagged at the N terminus with 3xFLAG with their native C-termini (WT), uncleavable mutants (GA) and mutants corresponding to the primed form with their active glycine exposed (G) (Figure 2(c)). When these constructs were expressed in wild-type HeLa cells treated with Torin1 and baf A1, both WT and G mutants underwent similar levels of lipidation (formation of band with lower apparent molecular weight) suggesting that priming of WT constructs occurred normally prior to their lipidation and did not dramatically affect the level of lipidation (Figure 2(d), **S2**). However, in *ATG4B* KO cells, WT constructs were present as bands with similar motility to GA mutants suggesting they were mainly unprocessed (Figure 2(d), **S2**). In these cells, small amounts of LC3A, GABARAP and GABARAPL1 lipidation were observed at high contrast for WT constructs, but at reduced levels compared to G mutants (Figure 2(d)). In this particular experiment, it was difficult to resolve the different forms of 3xFLAG-GABARAPL2.

Altogether, these results suggest that most LC3 subfamily isoforms absolutely require ATG4B for priming (LC3B, LC3B2 and LC3C). Conversely, priming of other LC3/GABARAP isoforms (LC3A and GABARAP subfamily) can occur in ATG4B-deficient cells but is markedly reduced in efficiency, thus limiting the amount of lipidated LC3/GABARAP that can accumulate.

### GABARAPL1 can localize to autophagic structures in cells lacking ATG4B

**Figure 3.**
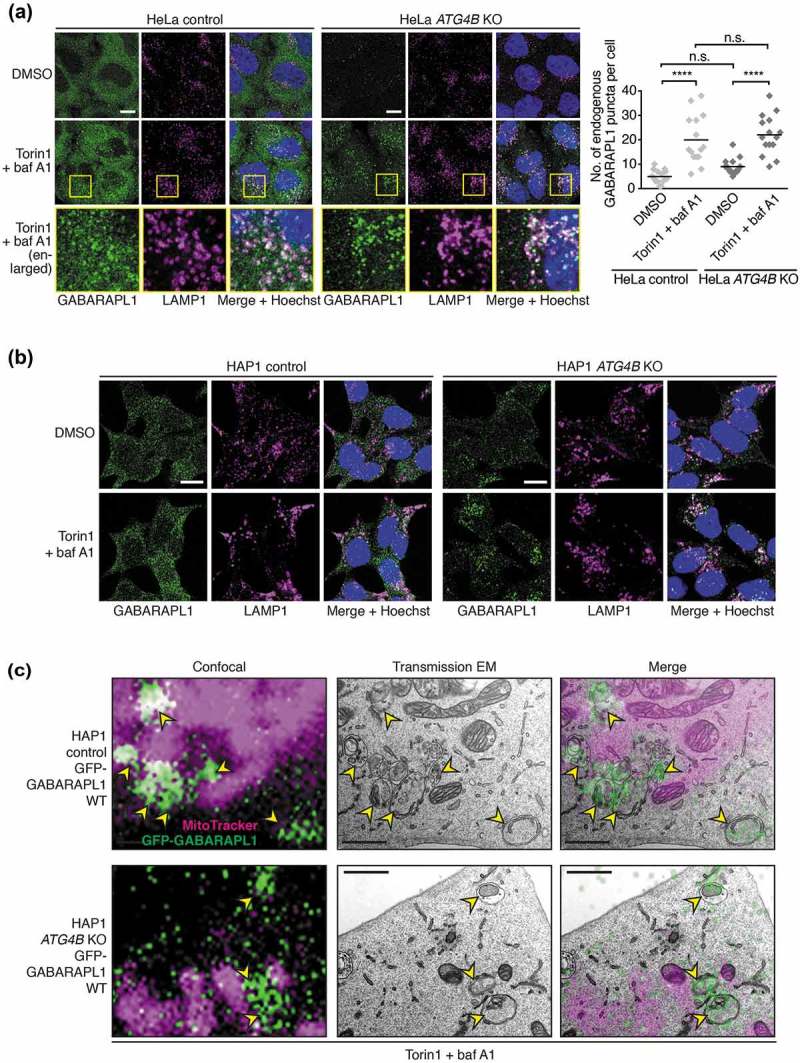
GABARAPL1 can localize to autophagic structures in the absence of ATG4B. (**a**) Immunocytochemistry of endogenous GABARAPL1 and LAMP1 in HeLa control or *ATG4B* KO cells, treated with DMSO or 250 nM Torin1 + 10 nM baf A1 for 3 h prior to fixation. Yellow boxes show region-of-interest in the Torin1 + baf A1 treated condition that is enlarged in the panels below. Scale bar: 10 µm. Quantification of GABARAPL1 puncta per cell shown on right hand side. n = 15 randomly-selected cells per condition. **** P ≤ 0.0001, n.s. (not significant) P > 0.05 (Sidak’s multiple comparison test). (**b**) Immunocytochemistry as described in Figure 3(a), for HAP1 control and *ATG4B* KO cells. (**c**) CLEM of GFP-GABARAPL1 WT stably expressed in in HAP1 control and *ATG4B* KO cells treated for 3 h with 250 nM Torin1 + 10 nM baf A1 prior to fixation. Yellow arrowheads indicate GFP-positive autophagic structures. Confocal microscopy image corresponds to a single confocal slice. Scale bar: 1 µm.

Following the observation of lipidation of GABARAP isoforms in response to autophagy stimulation in both wild-type and ATG4B-deficient cells, we decided to assess the subcellular localization of endogenous GABARAPL1 using a validated isoform-specific antibody [26]. We found that in both *ATG4B* KO HAP1 and HeLa cells, GABARAPL1 localized to distinct puncta that accumulated in an autophagy-dependent manner and colocalized with LAMP1 upon baf A1 treatment (Figure 3(a,b)), in stark contrast to the absence of LC3B puncta in *ATG4B* KO cells under the same conditions (Figure 1(a)). This is consistent with increased GABARAPL1 lipidation levels in response to treatment as observed by western blot (Figure 1(b)). Although in wild-type cells GABARAPL1 puncta were less pronounced due to the higher cytoplasmic levels of free GABARAPL1 (Figure 3(a,b)), quantitative image analysis revealed that both HeLa control and *ATG4B* KO cells formed similar numbers of basal and treatment-induced GABARAPL1 puncta (Figure 3(a)). To investigate whether these puncta truly represent autophagosomes, we performed correlative light and electron microscopy (CLEM) of wild-type and *ATG4B* KO HAP1 cells stably expressing GFP-GABARAPL1 (Figure 3(c)). We first confirmed that stably expressed GFP-GABARAPL1 WT (possessing its wild-type C terminus) could indeed localize to puncta in response to Torin1 and baf A1 treatment in both wild-type and *ATG4B* KO HAP1 cells (**Figure S3**). When GFP-GABARAPL1 puncta identified by light microscopy were located in transmission electron micrographs, they were seen to correspond to autophagic structures in both wild-type and *ATG4B* KO cells (Figure 3(c)). Altogether this shows that the remaining priming activity in *ATG4B* KO cells is sufficient to allow GABARAPL1 to localize to autophagic structures.

### Loss of ATG4B impairs autophagosome formation and SQSTM1 delivery to lysosomes

**Figure 4.**
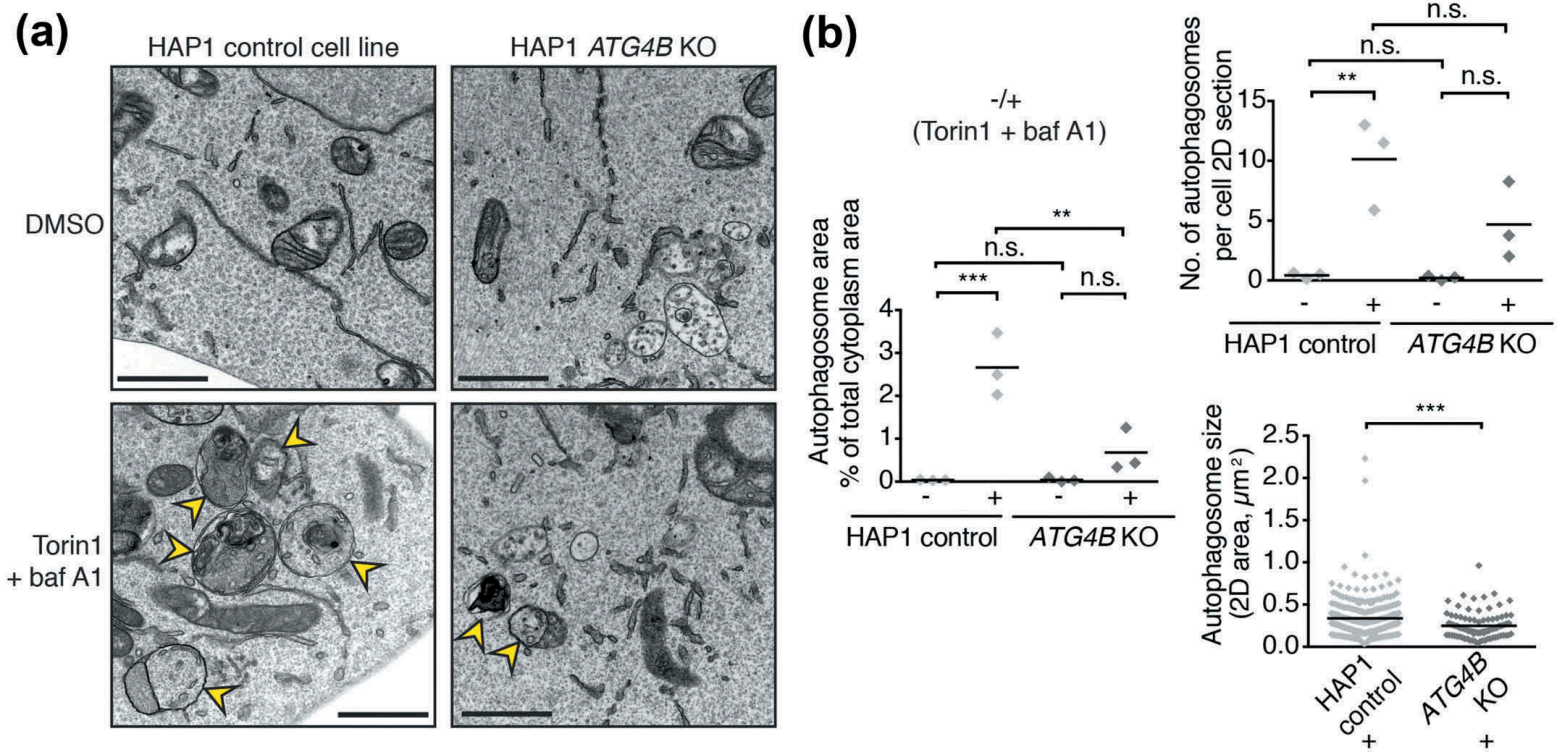
Autophagosome formation defect in cells lacking ATG4B. (**a**) Representative transmission electron microscopy (TEM) images of areas of cytoplasm in HAP1 control and *ATG4B* KO cells treated for 3 h with DMSO or 250 nM Torin1 + 10 nM baf A1 prior to fixation and sample processing. Yellow arrowheads indicate autophagic structures. Scale bar: 1 µm. (**b**) Quantitative TEM analysis of average cytoplasm area occupied by autophagic structures and average number of autophagic structures per cell from experiments represented in Figure 4(a). Data points show mean from each experiment (n = 3 independent experiments). In each experiment 8 randomly selected cell profiles were assessed per condition. *** P ≤ 0.001, ** P ≤ 0.01, n.s. (not significant) P > 0.05 (Sidak’s multiple comparison test). For quantification of autophagosome size in terms of 2D area, data points represent individual autophagic structures measured from Torin1- and baf A1-treated conditions pooled from 3 experiments (control n = 259, *ATG4B* KO n = 98). *** P ≤ 0.001, ** P ≤ 0.01 (Unpaired two-tailed t-test).

Having determined the impact of ATG4B loss on LC3/GABARAP priming and lipidation, we next decided to determine how this affects overall autophagosome formation. To achieve this, we performed transmission electron microscopy (TEM), a technique that allows the quantification of autophagosomes based on their distinct ultrastructure without relying on marker proteins. Using this method, autophagic structures can be identified by their characteristic morphology of cytoplasmic material sequestered within a double or multi-membrane compartments [27]. In untreated HAP1 control and *ATG4B* KO cells, autophagic structures were rarely observed (Figure 4(a,b)). Upon treatment with baf A1 and Torin1, a large number of autophagosomes was readily observed in control cells, occupying 2.66 ± 0.426% (mean ± SEM) of the total cytoplasm area. In treated *ATG4B* KO cells however, cytoplasm area occupied by autophagic structures was significantly reduced (0.677 ± 0.290%) (Figure 4(a,b)). However, autophagic structures were still observed in treated ATG4B-deficient cells and the number of structures per cell was not significantly reduced. These structures had an altered morphology, being significantly smaller than those seen in control cells. Autophagosomes in control cells were on average 655 ± 12.8 nm in diameter while structures in *ATG4B* KO cells had a mean diameter of 578 ± 17.7 nm (Figure 4(a,b)). Collectively, these data show that ATG4B is important for proper autophagosome formation but not absolutely required for the presence of autophagic structures.

**Figure 5.**
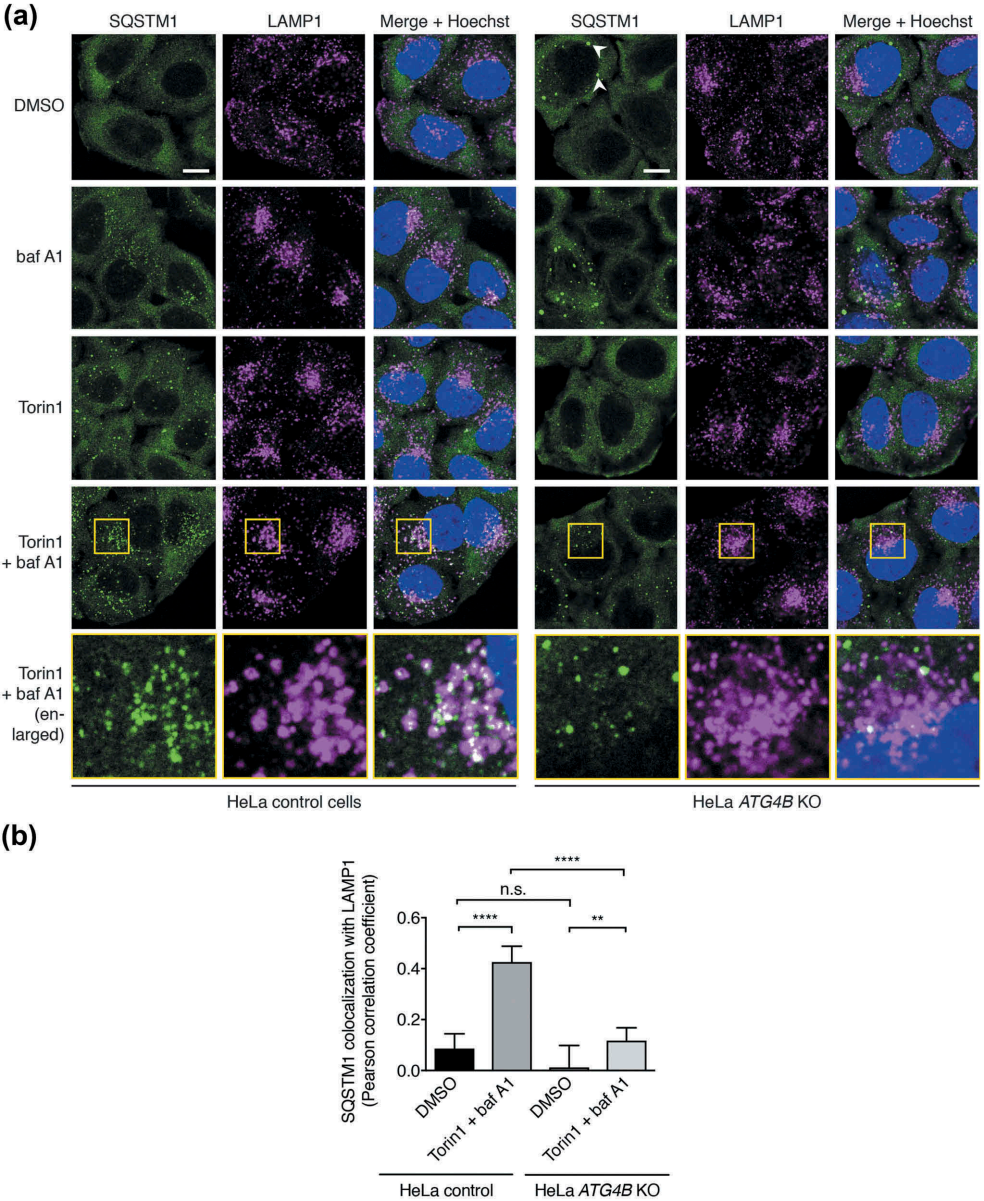
Impaired autophagic delivery of SQSTM1 to lysosomes in cells lacking ATG4B. (**a**) Immunocytochemistry of endogenous SQSTM1 and LAMP1 in control and *ATG4B* KO HeLa cells. Cells were treated for 3 h with DMSO or 250 nM Torin1 + 10 nM baf A1 prior to fixation and staining. White arrowheads indicate examples of large SQSTM1 bodies in *ATG4B* KO cells that likely correspond to protein aggregates. Yellow boxes show region-of-interest in the Torin1 + baf A1 treated condition that is enlarged in the panels below. Scale bar: 10 µm. (**b**) Assessment of colocalization between endogenous SQSTM1 and LAMP1 in HeLa control and *ATG4B* KO cells using single-plane confocal images as represented in Figure 5(a). Bars indicate mean and error bars show standard deviation (n = 10 randomly-selected cells per condition). **** P ≤ 0.0001, ** P ≤ 0.01, n.s. (not significant) P > 0.05 (Sidak’s multiple comparison test).

As an additional functional indicator of autophagic flux, we monitored the delivery of endogenous SQSTM1 to lysosomes marked by LAMP1 using immunofluorescence (Figure 5(a,b)). In wild-type HeLa cells, small SQSTM1 puncta accumulated within LAMP1-positive structures upon baf A1 treatment, consistent with a blockage in the autophagic degradation of SQSTM1 within autolysosomes. This accumulation was markedly increased in the additional presence of Torin1, consistent with an increase in delivery of SQSTM1 to lysosomes upon autophagic stimulation (Figure 5(a)). However in HeLa *ATG4B* KO cells, the recruitment of SQSTM1 to lysosomes upon Torin1 + baf A1 treatment was dramatically reduced (Figure 5(a,b)) and fewer small SQSTM1 puncta were observed under all treatment conditions (Figure 5(a)), signifying a diminished ability for these cells to recruit SQSTM1 to autophagosomes. Large SQSTM1 puncta that were typically negative for LAMP1 staining were observed in a subset of HeLa *ATG4B* KO cells under all treatment conditions (Figure 5(a), white arrowheads), likely corresponding to SQSTM1-positive protein aggregates that have previously been shown to accumulate in HeLa cells when autophagy is impaired [28]. We observed similar results in HAP1 cells during starvation by western blot (**Figure S4A**) and in response to compound treatment by immunocytochemistry (**Figure S4B**). These results are consistent with the SQSTM1 accumulation and reduced Torin1-induced degradation observed in *ATG4B* KO cells by western blot (Figure 1(b)). Altogether, loss of ATG4B impairs both the formation of autophagosomes and the recruitment of SQSTM1 to autophagosomes.

### Overexpression of pre-primed LC3B rescues autophagy defects in ATG4B KO cells

Next we decided to determine whether ATG4B plays an essential role in the autophagy pathway after executing pro-LC3B priming. To achieve this, we used a mutant form of LC3B that terminates at the C-terminal active glycine (G120) and thus bypasses the ATG4-dependent priming step and corresponds to primed/activated LC3B. Comparing the effects on autophagy of expression of LC3B-G120 in wild-type versus *ATG4B* KO cells should therefore reveal any autophagy defect caused specifically by the loss of ATG4B in steps subsequent to priming.

**Figure 6.**
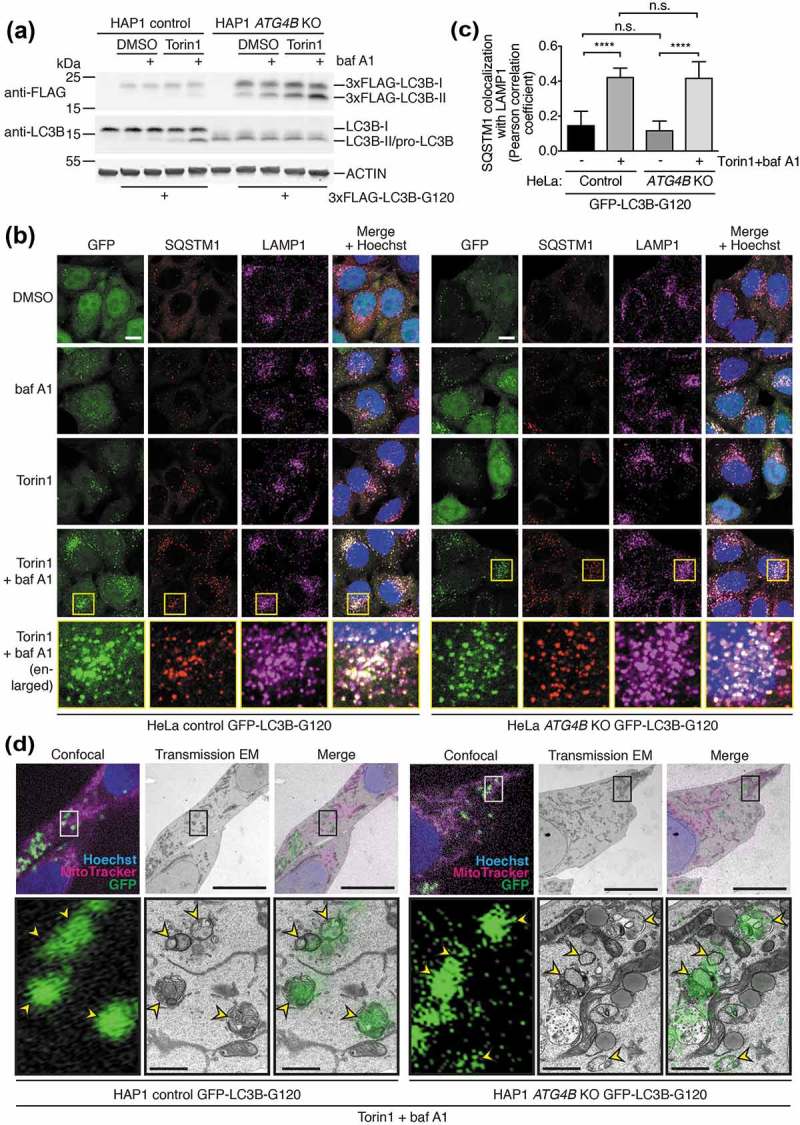
Pre-primed LC3B is lipidated, localizes to autophagosomes, and can restore endogenous SQSTM1 turnover when expressed at high levels in *ATG4B* KO cells. (**a**) HAP1 control and *ATG4B* KO cells transfected with 3xFLAG-LC3B-G120 using PEI were treated with combinations of DMSO or 250 nM Torin1 with or without 10 nM baf A1 for 3 h prior to lysis and western blot analysis. (**b**) Immunocytochemistry of endogenous SQSTM1 and LAMP1 in HeLa control and *ATG4B* KO cells stably expressing GFP-LC3B-G120 under control of the *CMV* promoter. Cells were treated for 3 h with combinations of DMSO or 250 nM Torin1 with or without 10 nM baf A1 for 3 h prior to fixation and staining. Yellow boxes show region-of-interest in the Torin1 + baf A1 treated condition that is enlarged in the panels below. Scale bar: 10 µm. (**c**) Assessment of colocalization between endogenous SQSTM1 and LAMP1 in HeLa control and *ATG4B* KO cells stably expressing *CMV* promoter-driven GFP-LC3B-G120 using single-plane confocal images as represented in Figure 6(b). Bars indicate mean and error bars show standard deviation (n = 10 randomly-selected cells per condition). **** P ≤ 0.0001, n.s. (not significant) P > 0.05 (Sidak’s multiple comparison test). (**d**) CLEM of *CMV*-driven GFP-LC3B-G120 stably expressed in HAP1 control and *ATG4B* KO cells. Cells were treated for 3 h with 250 nM Torin1 + 10 nM baf A1 prior to fixation. Upper panels show low magnification images with a boxed region-of-interest (ROI); scale bar: 10 µm. Lower panels show enlarged ROI, with yellow arrowheads indicating GFP-positive autophagic structures; scale bar: 1 µm. Confocal microscopy image corresponds to a single confocal slice.

When expressed in both wild-type and *ATG4B* KO HAP1 cells, 3xFLAG-LC3B-G120 underwent lipidation in an autophagy-dependent manner as determined by western blot, showing that bypassing priming can restore LC3B lipidation and flux in ATG4B-deficient cells (Figure 6(a). When the stably-expressed GFP-tagged version of this construct was examined by light microscopy, it was seen to localize to punctate structures that accumulated in a Torin1 and baf A1 treatment-dependent manner in both wild-type and *ATG4B* KO HeLa cells, in contrast to WT LC3B which could not form puncta in *ATG4B* KO cells (**Figure S5A**). When both HeLa (Figure 6(b)) and HAP1 cells (**Figure S5B**) stably expressing GFP-LC3B-G120 were stained for endogenous SQSTM1, this revealed that GFP puncta were positive for SQSTM1 in both wild-type and *ATG4B* KO genetic backgrounds. Under baf A1-treated conditions, these puncta also overlapped with LAMP1 staining (Figure 6(b), **S5B**). HeLa *ATG4B* KO cells expressing GFP-LC3B-G120 showed an identical pattern of SQSTM1 lysosome delivery to control cells, because there was no significant difference in the extent of SQSTM1 colocalization with LAMP1 between the two cell lines, both in the absence of treatment and when elevated in response to Torin1 + baf A1 treatment (Figure 6(c)). Therefore, GFP-LC3B-G120 expression in *ATG4B* KO cells could efficiently rescue the defect in SQSTM1 delivery to lysosomes observed in untransduced cells (Figure 5(a,b), **S4B**). When puncta of stably expressed GFP-LC3B-G120 in HAP1 cells were examined by CLEM, they were seen to correspond to autophagic structures in both wild-type and ATG4B-deficient cells (Figure 6(d)), suggesting that ATG4B is not required for the correct localization of LC3B after priming has occurred.

Because ATG4-mediated delipidation has previously been implicated in autophagosome closure, we next assessed whether LC3B-positive vesicles formed in ATG4B-deficient cells represent fully sealed structures using a protease protection assay (**Figure S5C**). In this assay, unpermeabilized cell homogenates and an equivalent permeabilized sample are subject to trypsin treatment followed by western blot analysis. Proteins that are specifically protected from degradation in the unpermeabilized sample but become sensitive to degradation in the presence of detergent are considered to be protected by membrane and thus localized within sealed vesicles or organelles. This assay was able to distinguish between proteins on the inside and outside of a sealed double-membrane organelle, since the mitochondrial matrix protein PDHA1 (pyruvate dehydrogenase E1 alpha 1 subunit) was only efficiently degraded by trypsin in the presence of detergent, while the cytosol-facing outer mitochondrial membrane protein MFN2 (mitofusin 2) was degraded completely in both presence and absence of detergent. The lysosome membrane protein LAMP1 served as a loading control in this assay since it was relatively resistant to trypsin. We found that a similar fraction of transiently transfected 3xFLAG-LC3B-G120 was resistant to trypsin degradation in the absence of detergent in both HAP1 control and *ATG4B* KO cells treated with Torin1 and baf A1 (**Figure S5C**). This suggests that closure of autophagosomes is not impaired in the absence of ATG4B-mediated LC3B delipidation.

Altogether, we find that bypassing pro-LC3B processing by high expression of pre-primed LC3B rescues the autophagy defect caused by loss of ATG4B and does not produce any detectable abnormalities in LC3B localization, SQSTM1 degradation or autophagosome closure. This suggests that ATG4B does not play an essential role in autophagy downstream of LC3/GABARAP priming.

### Combined loss of ATG4A/4B does not block priming of endogenous GABARAPL1/L2 or impair rescue of autophagy by high-level overexpression of pre-primed LC3/GABARAP.

**Figure 7.**
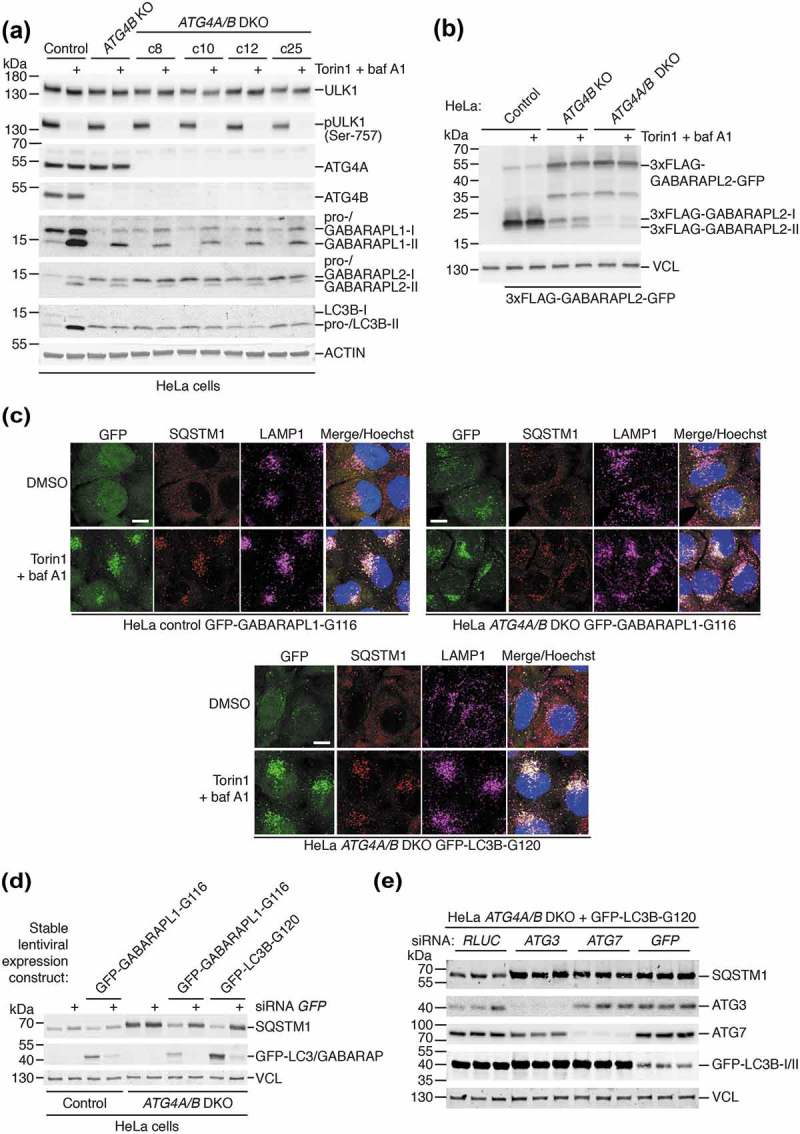
Additional loss of ATG4A in *ATG4B* KO cells only partially further reduces GABARAP subfamily priming and does not prevent rescue of autophagy by pre-primed LC3/GABARAP. (**a**) HeLa control, *ATG4B* KO and different *ATG4A/B* double knockout (DKO) clones were treated for 3 h with DMSO or 250 nM Torin1 + 10 nM baf A1 prior to lysis and western blot analysis. See also Figure S6A for analysis of the same clones by immunocytochemistry of endogenous GABARAPL1. (**b**) HeLa control, *ATG4B* KO and *ATG4A/B* DKO (c25) were transiently transfected with 3xFLAG-GABARAPL2-GFP. After 24 h, cells were treated for 3 h with DMSO or 250 nM Torin1 + 10 nM baf A1 prior to lysis and western blotting. 3xFLAG-GABARAPL2-GFP was detected using anti-FLAG antibody. (**c**) HeLa cells stably transduced with GFP-GABARAPL1-G116 (Control and *ATG4A/B* DKO c25) and GFP-LC3B-G120 (*ATG4A/B* DKO c25) were treated for 3 h with DMSO or 250 nM Torin1 + 10 nM baf A1 prior to fixation and immunocytochemistry to reveal endogenous SQSTM1 and LAMP1 localization. Scale bar: 10 µm. See also Figure S6B for immunocytochemistry of untransduced cells. (**d**) Untransduced and stably-transduced HeLa control and *ATG4A/B* DKO (c25) cells were assessed for SQSTM1 protein level by western blotting, 48 h after transfection with siRNA targeting *RLUC* as a negative control (lanes without +), or *EGFP* (lanes with +). GFP-LC3B-G120 and GFP-GABARAPL1-G116 were detected using anti-GFP antibody. (**e**) HeLa *ATG4A/B* DKO (c25) cells stably expressing GFP-LC3B-G120 were transfected with siRNA targeting *RLUC* (negative control), *ATG3, ATG7* or *GFP* (positive control). After 48 h, cells were lysed and subject to western blot analysis to assess SQSTM1 protein level. GFP-LC3B-G120 was detected using anti-GFP antibody.

Following the discovery that ATG4B is not essential for GABARAP subfamily priming or autophagy downstream of LC3B priming, we next sought to identify the ATG4 isoforms that compensate for loss of ATG4B. Because ATG4A is the only other isoform known to efficiently process GABARAP isoforms *in vitro*, this was the strongest candidate for being responsible for GABARAP isoform function in ATG4B-deficient cells. To address the role of ATG4A, we generated multiple *ATG4A/B* double knockout (DKO) HeLa cell clones using CRISPR-Cas9 and validated loss of ATG4A expression by western blotting (Figure 7(a)). We found that when each of these cell clones were treated with Torin1 and baf A1, they could all still form lipidated GABARAPL1 and GABARAPL2 (Figure 7(a)) in addition to being able to form endogenous GABARAPL1 puncta (**Figure S6A**), although the levels of lipidation and puncta formation appeared to be slightly reduced compared to *ATG4B* KO cells. When 3xFLAG-GABARAPL2-GFP was expressed in HeLa *ATG4A/B* DKO cells, it showed a marked reduction in priming compared to *ATG4B* KO cells (Figure 7(b)). Altogether, this shows that ATG4A is also involved in priming of GABARAPL1 and GABARAPL2 but is not the sole ATG4 isoform responsible for priming in ATG4B-deficient cells.

We confirmed by immunocytochemistry that *ATG4A/B* DKO HeLa cells are impaired in SQSTM1 delivery to lysosomes (**Figure S6B**), showing the same defect as *ATG4B* KO cells (Figure 5(a)). Stable expression of both GFP-LC3B-G120 and the equivalent bypass mutant of GABARAPL1 (GFP-GABARAPL1-G116) in *ATG4A/B* DKO HeLa cells could rescue SQSTM1 delivery to LAMP1-positive structures (Figure 7(c)). Rescue of basal SQSTM1 turnover could also be observed in these cells, since by western blot we could detect reduced levels of SQSTM1 similar to those in control cells, in contrast to untransduced *ATG4A/B* DKO cells which exhibited strongly elevated basal levels of SQSTM1 (Figure 7(d)). This rescue of basal SQSTM1 turnover was specifically due to bypass construct expression, because knockdown of the bypass construct using siRNA targeting *GFP* resulted in an elevation of SQSTM1 expression levels approaching that seen in untransduced *ATG4A/B* DKO cells (Figure 7(d)). Further, rescue of SQSTM1 turnover by GFP-LC3B-G120 expression in *ATG4A/B* DKO cells was dependent on LC3/GABARAP lipidation and autophagy, since suppression of ATG3 and ATG7 in these cells led to a strong accumulation of SQSTM1 similar to the effect of GFP knockdown (Figure 7(e)). Collectively these data suggest that both ATG4A and ATG4B are dispensable for basal and Torin1-induced autophagy downstream of LC3/GABARAP priming.

We also separately generated HeLa *ATG4A/B* DKO cells stably expressing GFP-LC3B-G120 or GFP-GABARAPL1 under control of a weak (*PGK*) promoter, to determine whether expressing bypass mutants at lower levels would be sufficient to rescue autophagy (**Figure S7A**). These cells could form GFP puncta with the expected localization pattern (**Figure S7B**), however they were notably less efficient at reducing basal SQSTM1 level **(Figure S7A**) and rescuing SQSTM1 delivery to lysosomes (**Figure S8**) compared to equivalent *ATG4A/B* DKO cells expressing higher levels of GFP-LC3B-G120 under control of the *CMV* promoter. These results argue that the supply of primed LC3/GABARAP (i.e. through recycling) might be a stronger requirement for autophagy than ATG4 delipidation activity *per se*. Establishing whether delipidation by ATG4 isoforms is necessary for functional aspects of autophagy beyond regulating the level of primed LC3/GABARAP (e.g. autophagosome-lysosome fusion) requires a model system in which primed LC3/GABARAP supply is plentiful. Therefore, we continued characterizing ATG4-deficient cells expressing *CMV* promoter-driven levels of bypass mutants for the remainder of our study.

### ATG4C and ATG4D both additionally contribute to GABARAP isoform priming and lipidation independent of CASP3 expression.

**Figure 8.**
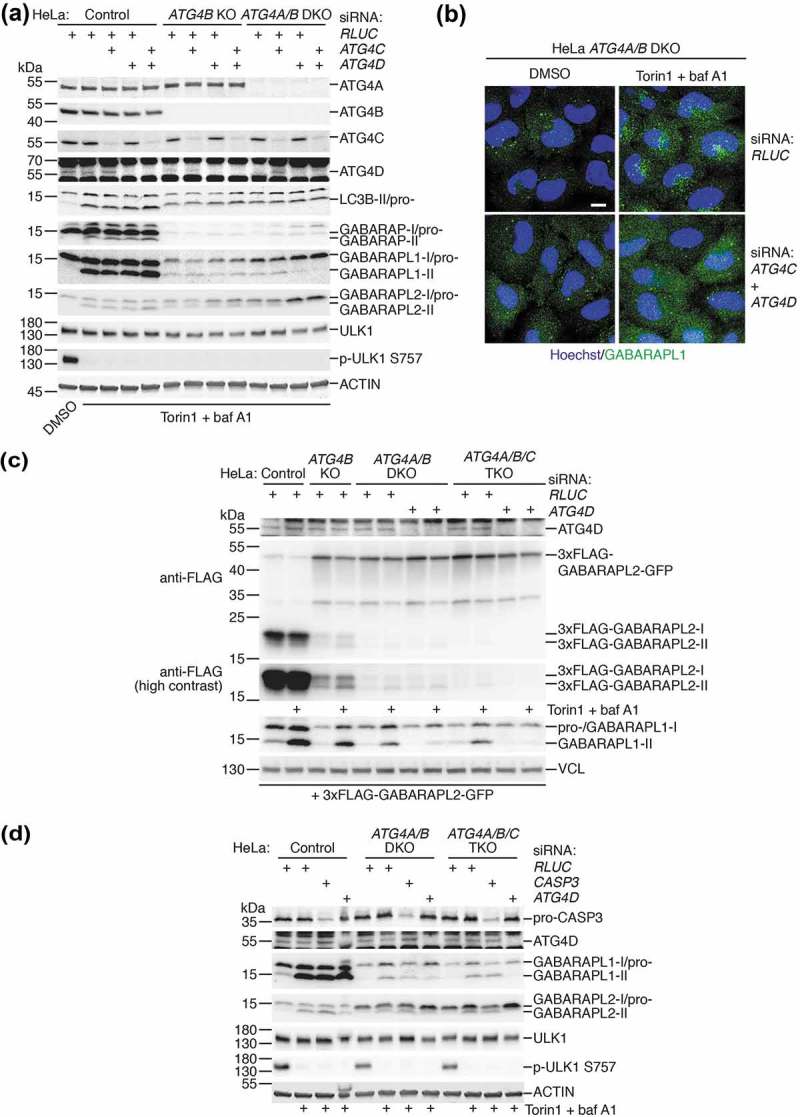
All ATG4 isoforms contribute to GABARAPL1 and GABARAPL2 priming and lipidation independently of CASP3 expression. (**a**) HeLa control, *ATG4B* KO and *ATG4A/B* DKO (c25) cells transfected with siRNA targeting *ATG4C, ATG4D* or *RLUC* (negative control) were treated for 3 h with DMSO or 250 nM Torin1 + 10 nM baf A1 prior to lysis and western blot analysis. (**b**) Immunocytochemistry of endogenous GABARAPL1 (in green, with Hoechst staining shown in blue) in HeLa *ATG4A/B* DKO (c25) cells treated for 3 h with DMSO or 250 nM Torin1 + 10 nM baf A1, after transfection with siRNA against *RLUC* (negative control) or *ATG4C* + ATG4D. Scale bar: 10 µm. (**c**) HeLa control, *ATG4B* KO, *ATG4A/B* DKO (c25) and *ATG4A/B/C* triple knockout (TKO, c22) cells were transfected with siRNA against either *RLUC* or *ATG4D* prior to transfection with 3xFLAG-GABARAPL2-GFP. Cells were treated for 3 h with DMSO or 250 nM Torin1 + 10 nM baf A1 prior to lysis and western blot analysis. See also Figures S9A-C for additional characterization of HeLa *ATG4/A/B/C* TKO cells. (**d**) Western blotting of HeLa control, *ATG4A/B* DKO (c25) and *ATG4A/B/C* TKO (c22) cell lysates following siRNA knockdown of CASP3 or ATG4D. Cells were treated for 3 h with DMSO or 250 nM Torin1 + 10 nM baf A1 prior to lysis.

Next, to identify the ATG4 isoforms responsible for residual GABARAP isoform priming in *ATG4A/B* DKO cells, we used RNAi to deplete combinations of ATG4C and ATG4D in different HeLa cell lines treated with Torin1 and baf A1 to induce GABARAP isoform lipidation. As seen in Figure 8(a), RNAi-mediated suppression of ATG4C and ATG4D expression in HeLa *ATG4A/B* DKO cells was sufficient to completely block lipidation of GABARAPL1 and GABARAPL2 compared to control RNAi. In these cells, loss of ATG4D alone produced a stronger inhibition of GABARAPL1/L2 lipidation compared to ATG4C loss, suggesting that ATG4D was the main isoform involved in residual priming of these GABARAP isoforms. Nonetheless, the observation that loss of both ATG4C and ATG4D produced a complete inhibition of lipidation suggests a partial involvement of ATG4C in GABARAPL1/L2 priming. Importantly, these effects could not be observed in HeLa cells lacking ATG4B alone, confirming that ATG4A is also involved in endogenous GABARAPL1 and GABARAPL2 priming. We confirmed these findings by knockdown of ATG4C and ATG4D in HeLa *ATG4A/B* DKO cells prior to immunostaining for endogenous GABARAPL1 (Figure 8(b)). This revealed that additional loss of ATG4C and ATG4D blocked the formation of small perinuclear GABARAPL1-positive puncta that normally accumulated in the presence of Torin1 and baf A1 in *ATG4A/B* DKO cells. We then determined that expression of ATG4D alone was sufficient for priming and lipidation of GABARAPL1 and GABARAPL2, since *ATG4A/B/C* triple knockout (TKO) HeLa cells generated by CRISPR-Cas9 (**Figure S9A**) also retained the ability to form lipidated GABARAPL1 and GABARAPL2 (**Figure S9B**) and GABARAPL1-positive puncta in response to treatment (**Figure S9C**).

To observe the effect of loss of each individual ATG4 isoform on GABARAPL2 priming, we expressed 3xFLAG-GABARAPL2-GFP in each of the generated HeLa cell lines following transfection with siRNA (Figure 8(c)). This revealed that loss of each additional ATG4 isoform led to a reduction in priming, but that a complete loss of priming could only be observed upon loss of all ATG4 isoforms. This effect could also be observed for endogenous GABARAPL1 in the same cell lysates, assuming that loss of lipidated GABARAPL1 in response to treatment was a result of impaired priming (Figure 8(c)). Together these data show that all human ATG4 isoforms contribute to the priming of LC3/GABARAP isoforms, because complete loss of LC3/GABARAP priming and lipidation in cells could only be achieved upon loss of all ATG4 isoforms.

Because CASP3 promotes ATG4D activity *in vitro* [18], we performed knockdown of CASP3 by RNAi to determine whether the residual activity of ATG4C and ATG4D detected in HeLa cells lacking ATG4A/B was dependent on CASP3 expression (Figure 8(d)). Despite efficient knockdown, we could not detect a reduction in treatment-induced GABARAPL1 or GABARAPL2 lipidation caused by CASP3 suppression in *ATG4A/B* DKO and *ATG4A/B/C* TKO cells, in contrast to knockdown of ATG4D. Therefore, the residual priming activity of ATG4C and ATG4D detected in HeLa cells lacking ATG4A/B does not appear to be dependent on CASP3 expression.

### Delipidation by all ATG4 isoforms is not essential for autophagosome-lysosome fusion or preventing LC3/GABARAP mislocalization.

Finally, we decided to determine if delipidation by any ATG4 isoform plays an important role in aspects of autophagy beyond LC3/GABARAP recycling. Since we already established that ATG4A/B-mediated delipidation is not required for autophagosome closure and lysosome fusion when primed LC3/GABARAP is expressed at high levels (Figures 6 and 7(c,d)), we decided to investigate the consequences of further depletion of ATG4C and ATG4D in the same scenario.

**Figure 9.**
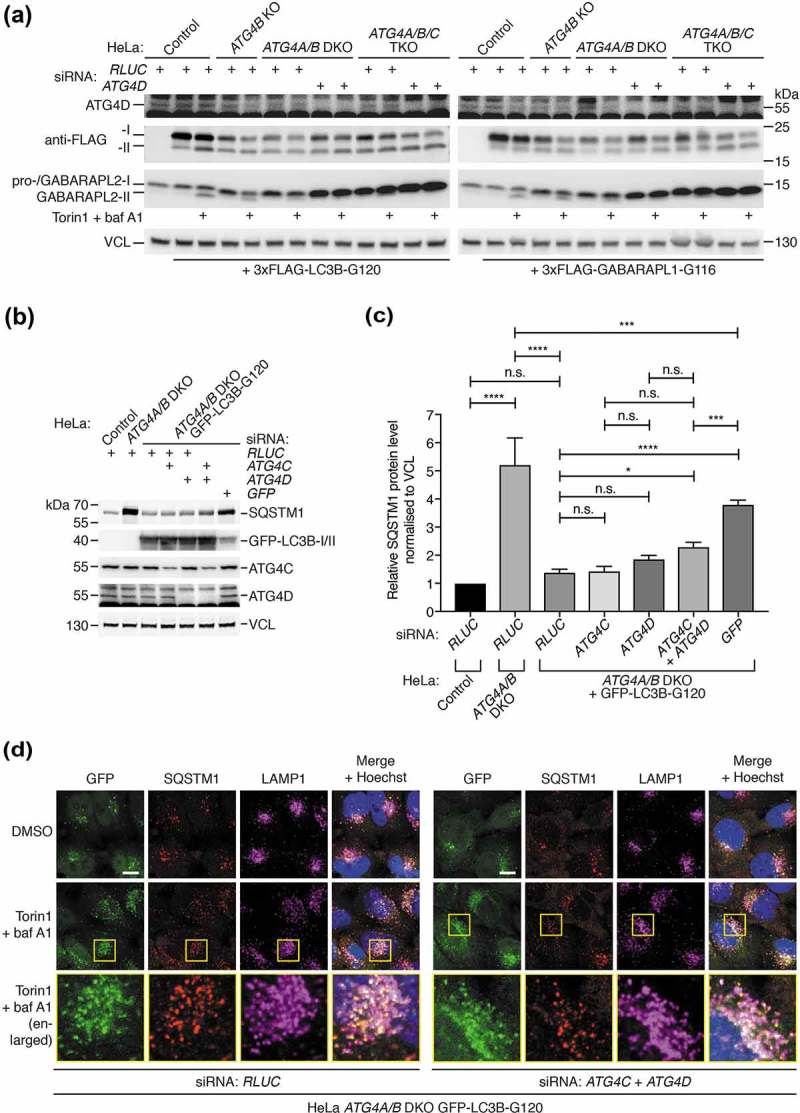
Delipidation by ATG4 isoforms is not essential for autophagy of SQSTM1 upon high expression of pre-primed LC3B. (**a**) HeLa control, *ATG4B* KO, *ATG4A/B* DKO (c25) and *ATG4A/B/C* triple knockout (TKO, c22) were transfected with siRNA against either *RLUC* or *ATG4D* prior to transfection with 3xFLAG-LC3B-G120 or 3xFLAG-GABARAPL1-G116. Cells were treated for 3 h with DMSO or 250 nM Torin1 + 10 nM baf A1 prior to lysis and western blot analysis. Anti-FLAG antibody was used to assess the lipidation status of 3xFLAG-LC3B-G120 and 3xFLAG-GABARAPL1-G116. (**b**) HeLa control, *ATG4A/B* DKO (c25) and *ATG4A/B* DKO (c25) cells stably expressing GFP-LC3B-G120 were transfected with combinations of siRNA targeting *RLUC* (negative control) *ATG4C, ATG4D* or *GFP* (positive control) prior to lysis and western blot analysis to assess the impact of knockdown on basal SQSTM1 protein level. (**c**) Quantification of SQSTM1 protein level normalized to VCL protein level from knockdown experiments represented in Figure 9(b). Bars indicate mean and error bars show standard deviation (n = 4 independent experiments). **** P ≤ 0.0001, *** P ≤ 0.001, * P ≤ 0.05, n.s. P > 0.05 (Sidak’s multiple comparison test). (**d**) Immunocytochemistry of endogenous SQSTM1 and LAMP1 in HeLa *ATG4A/B* DKO (c25) cells stably expressing GFP-LC3B-G120 and transfected with siRNA targeting *RLUC* or *ATG4C* + *ATG4D*. Cells were treated for 3 h with DMSO or 250 nM Torin1 + 10 nM baf A1 for 3 h prior to fixation and staining. Yellow boxes show region-of-interest in the Torin1 + baf A1-treated condition that is enlarged in the panels below. Scale bar: 10 µm.

When 3xFLAG-tagged LC3B-G120 and GABARAPL1-G116 were expressed in HeLa cells lacking different ATG4 isoforms (Figure 9(a)), we noted that combined loss of ATG4 isoforms did not have a dramatic effect on the bypass construct lipidation levels, despite priming of endogenous GABARAPL2 being efficiently blocked when all ATG4 isoforms were suppressed. This is in stark contrast to the equivalent experiment in yeast, where complete loss of Atg4 activity results in the entire pool of bypass mutant Atg8 becoming lipidated [10–12]. Therefore ATG4-mediated delipidation is not necessary for separate pools of free and lipidated LC3/GABARAP to exist in mammalian cells, although it must be noted that this was determined when primed LC3/GABARAP was overexpressed, and technical limitations prevent us from establishing whether the same is true at endogenous expression levels of primed LC3/GABARAP. Nonetheless, we were unable to identify a particular ATG4 isoform that, when suppressed, could drastically increase the lipidation levels of overexpressed bypass mutant LC3/GABARAP.

To determine whether delipidation by any ATG4 isoform is important for basal autophagy, we monitored endogenous SQSTM1 protein level in *ATG4A/B* DKO HeLa cells stably expressing high levels of GFP-LC3B-G120 following siRNA-mediated knockdown of ATG4C and ATG4D (Figure 9(b,c)). As described above, stable expression of *CMV* promoter-driven GFP-LC3B-G120 in *ATG4A/B* DKO HeLa cells efficiently rescues the defect in endogenous SQSTM1 turnover. When quantified from 4 experiments (Figure 9(c), representative experiment shown in Figure 9(b)), loss of ATG4A/B resulted in a 5.23 ± 0.95 (mean ± SEM) fold increase in basal SQSTM1 protein level relative to wild-type control cells (P < 0.0001). Upon stable expression of GFP-LC3B-G120 in *ATG4A/B* DKO cells, SQSTM1 protein reverted to a level that was not significantly different to wild-type cells (1.39 ± 0.12, P = 0.890). Knockdown of the rescue construct using siRNA targeting *GFP* dramatically increased SQSTM1 protein level to 3.80 ± 0.16 (P < 0.0001, versus SQSTM1 level of 1.39 ± 0.12 in equivalent *RLUC* siRNA-transfected cells), signifying a disruption in autophagy. In contrast, levels of SQSTM1 upon knockdown of ATG4C (1.43 ± 0.16) or ATG4D (1.86 ± 0.13) alone were not significantly raised (P > 0.999 and P = 0.702 respectively, versus equivalent *RLUC* siRNA-transfected cells). Only upon combined knockdown of ATG4C and ATG4D was there a mild increase in SQSTM1 level (2.30 ± 0.16, P = 0.0337 versus equivalent *RLUC* siRNA-transfected cells) and this was substantially less that the raised level of SQSTM1 upon GFP knockdown (P = 0.0002, SQSTM1 level of 3.80 ± 0.16). From this, we conclude that delipidation by ATG4 isoforms is not essential for basal autophagy when primed LC3B is overexpressed. If the opposite were true, we would expect to observe a much more dramatic increase in SQSTM1 level upon loss of all ATG4 isoforms in cells stably expressing GFP-LC3B-G120, e.g. similar to the effect of GFP knockdown.

We also examined the localization of endogenous SQSTM1 in response to Torin1 and baf A1 treatment in HeLa *ATG4A/B* DKO cells stably expressing GFP-LC3B-G120 following knockdown of ATG4C and ATG4D (Figure 9(d)). This revealed that GFP- and SQSTM1 double-positive structures can accumulate in response to treatment and localize to LAMP1-positive lysosomes despite loss of all ATG4 delipidation activity, overall suggesting that delipidation by ATG4 isoforms is not functionally required for autophagosome-lysosome fusion.

Lastly, we decided to determine whether ATG4-mediated delipidation was necessary to prevent mislocalization of LC3/GABARAP, since this is known to be an important function of Atg4 in yeast [10–12]. We assessed the basal localization of GFP-LC3B-G120 and GFP-GABARAPL1-G116 stably expressed at high levels in wild type HeLa cells compared to cells lacking all ATG4 isoforms (**Figure S10**). Upon loss of ATG4-mediated delipidation, we were unable to detect any change in the localization of GFP-LC3B-G120 or GFP-GABARAPL1-G116 in relation to the endoplasmic reticulum, early endosomes, Golgi or lysosomes. We noted a partial Golgi localization of GFP-GABARAPL1-G116, but this was also observed in HeLa control cells and was therefore unrelated to delipidation.

Together we conclude that while all ATG4 isoforms contribute to the essential priming of LC3/GABARAP, delipidation by ATG4 isoforms (when primed LC3/GABARAP supply is plentiful) does not play a critical functional role in autophagosome-lysosome fusion and preventing LC3/GABARAP mislocalization in human cells.

## Discussion

Determining Atg gene isoform redundancy is of crucial importance for understanding the effect of autophagy on mammalian development. It is also an important consideration in the design and validation of novel therapeutic strategies for modulating autophagy. In the human autophagy LC3–PE conjugation system, there are seven LC3/GABARAP isoforms that are processed by four ATG4 isoforms. However, the requirement of ATG4 isoforms for processing of LC3/GABARAP isoforms in human cells has remained uncharacterized to date. Previous reports into ATG4 function have been limited to the study of individual ATG4 isoforms or performed using recombinant proteins or ectopic gene overexpression. The recent development of CRISPR-Cas9 technology for genome editing has greatly accelerated the ability to perform loss-of-function studies of combinations of endogenous genes in cells. This approach was recently used to dissect LC3/GABARAP isoform function [14], and we have now adopted a similar strategy to determine the redundancy of ATG4 isoforms in autophagy.

By characterizing *ATG4B* KO cells, we found that ATG4B was required for the priming of most LC3 subfamily isoforms (including LC3B and LC3C) but a reduced level of priming of GABARAP subfamily isoforms could still occur in ATG4B-deficient cells. Using this cell model in combination with biochemical assays and expression of mutant LC3/GABARAP constructs, we were also able to determine the relative SDS-PAGE migration of all forms of human LC3/GABARAP, building on previous discoveries [23]. Based on *in vitro* data, we initially predicted that all LC3/GABARAP processing would be executed by ATG4A and ATG4B under basal and autophagy-induced cellular conditions. As such, we were surprised that combined loss of ATG4A/B did not appear have a major effect on endogenous GABARAPL1/L2 processing compared to loss of ATG4B alone. This led to our discovery that ATG4C and ATG4D also play a role in cells, since loss of all ATG4 isoforms was required to completely block the processing of all LC3/GABARAP isoforms. Importantly, the remaining processing activity in *ATG4A/B* DKO and *ATG4A/B/C* TKO cells was not affected by a reduction in CASP3 expression, despite ATG4C and ATG4D being reported to have little detectable processing activity *in vitro* unless activated by CASP3 [16,18]. It is important to note that in our study, we did not address whether induction of CASP3 activity could enhance the remaining endogenous ATG4D activity in *ATG4A/B/C* TKO cells, as might be predicted based on previous data [18]. There are several factors that could potentially contribute to the disparity between *in vitro* LC3/GABARAP processing and endogenous processing in cells. These include differences in protease and substrate concentration, reaction time, post-translational modifications, and the effect of protein tags in synthetic constructs. Our data is consistent with ATG4B being the functionally dominant isoform since its loss results in a strong autophagy and LC3/GABARAP processing defect, but contributions to LC3/GABARAP lipidation by all other ATG4 isoforms could nonetheless be detected upon loss of ATG4B. We suggest that the broad redundancy of ATG4 isoforms might explain the viability of ATG4B-deficient mice [19] in contrast to mice lacking ATG3 [20]. Following submission of our article, a related study reported using CRISPR-Cas9 to generate HEK293 cells lacking all four ATG4 isoforms [29]. Interestingly, it was shown that rescue of these cells with ectopic ATG4C or ATG4D could restore mild lipidation of GABARAPL1 [29], which is consistent with our data showing broad ATG4 isoform redundancy in GABARAP subfamily priming using a sequential knockout/knockdown strategy.

Our study is the first of its kind to begin addressing the key question of whether ATG4-mediated delipidation of LC3/GABARAP plays an essential role in mammalian autophagy. We discovered that expressing high levels of primed LC3B or GABARAPL1 in cells lacking ATG4A/B could restore the defect in autophagic SQSTM1 degradation. This suggests that the autophagy defect in ATG4A/B-deficient cells arises due to an insufficient supply of primed LC3/GABARAP. Interestingly, rescue of *ATG4A/B* DKO cells with reduced levels of primed LC3B or GABARAPL1 driven by the *PGK* promoter was not sufficient to restore autophagy. This suggests that delipidation may be important for LC3/GABARAP recycling. Alternatively, it could be that factors unrelated to delipidation prevent lower expression of a single primed LC3/GABARAP isoform from being to able to rescue the autophagy defect of *ATG4A/B* DKO cells, such as: competition of bypass mutants with endogenous pro-LC3/GABARAP, insufficient expression levels of the total pool of primed LC3/GABARAP (which would normally consist of up to seven isoforms in wild-type cells), and possible non-redundant functions of single LC3/GABARAP isoforms that might only be apparent at low expression levels. Technical challenges prevent us from being able to generate a model addressing the role of delipidation while controlling for these other factors (e.g. *ATG4* KO cells expressing endogenous levels of all primed LC3/GABARAP isoforms that are resistant to known destabilizing effects of ATG4B loss, lacking all endogenous pro-LC3/GABARAP). Therefore, extensive further studies are necessary to understand the contrasting results observed when using different expression levels of LC3/GABARAP bypass mutants in our *ATG4A/B* DKO cell rescue experiments.

We further suppressed the function of ATG4C and ATG4D in *ATG4A/B* DKO cells expressing high levels of GFP-LC3B-G120 to determine if remaining ATG4 isoforms played an essential role in delipidation during autophagy, independent of any possible recycling function. We could only observe a relatively modest increase in basal SQSTM1 level for cells stably expressing active LC3B when all ATG4 isoforms were lost. It is possible that this mild effect could be related to differences in endogenous GABARAP subfamily isoform function rather than delipidation, since cells lacking all ATG4 isoforms show a complete absence of primed endogenous LC3/GABARAP compared to *ATG4A/B* DKO cells that possess some endogenous GABARAP isoform function. Further, we could observe evidence of autophagosome-lysosome fusion in cells lacking delipidation by all ATG4 isoforms. Therefore, our data suggest that delipidation by ATG4 isoforms is not required for autophagy in mammalian cells aside from any potential role in primed LC3/GABARAP supply. It could be the case that other enzymes exist that can remove LC3/GABARAP from membranes, or alternatively the removal of LC3/GABARAP from the surface of autophagosomes might not be required for autophagy to progress.

Although it is well established that ATG4 can cleave lipidated LC3 *in vitro* [9,24] and it has been suggested that very little LC3 is detected on the outer membrane of autolysosomes [30,31], there is little experimental data to link these observations and directly implicate ATG4 in the delipidation of LC3 *in vivo*. There are technical challenges associated with obtaining such evidence. It has long been known that overexpression of catalytically-inactive ATG4B in cells is sufficient to impair LC3 lipidation through binding and sequestering free LC3 [32]. Therefore it can be presumed that any effects of ATG4B depletion on the localization or amount of lipidated LC3 may not necessarily be dependent on ATG4B activity. Recent research has uncovered at least two distinct LIR-dependent binding interactions between ATG4 and LC3 [25,33]. These could serve as additional non-catalytic mechanisms by which ATG4 could negatively regulate LC3 lipidation, however mutations that disrupt these interactions also severely impede the catalytic activity of ATG4 [25,33]. Consequently, it is currently not possible to distinguish between catalytic and non-catalytic events mediated by ATG4 that occur after LC3/GABARAP priming, since one mechanism cannot yet be experimentally perturbed without affecting the other. Post-translational modifications add a further layer of complexity to the regulation of LC3 processing by ATG4 *in vivo*. We and others have recently described a mechanism by which the upstream kinase ULK1 (Atg1 in yeast) can phosphorylate ATG4B and inhibit both its activity and binding affinity towards LC3 in cells [21,34]. Combined with our findings, these insights should lead to a re-assessment of the role of ATG4 downstream of LC3/GABARAP priming, with the ultimate aim to distinguish between binding and delipidation effects while accounting for the contributions of different ATG4 and LC3/GABARAP isoforms.

Our study has uncovered important technical considerations for future studies of ATG4 and LC3/GABARAP. Firstly, we found that levels of lipidated LC3/GABARAP detected by western blot could be underestimated due to instability arising from ATG4 activity in cell lysates. When endogenous or overexpressed ATG4 is present in cell lysates, it is capable of converting pro- or lipidated LC3/GABARAP to the free form. This phenomenon originally resulted in us coming to the false conclusion that lipidated GABARAP isoforms were more abundant in ATG4B-deficient cells, when this was actually an artifact arising from higher ATG4 activity in the lysates of wild-type cells compared to *ATG4B* KO cells. Therefore when studying LC3/GABARAP lipidation in cells by western blotting it is recommended to include NEM or a denaturant in the lysis buffer in order to immediately inactivate ATG4 isoforms following lysis and thus avoid detecting non-physiological effects on LC3/GABARAP processing. Secondly, we confirm a previous study reporting that human pro-LC3B has a migration rate similar to LC3B-II in SDS-PAGE [23] by demonstrating that this is true for endogenous LC3B in human cells completely lacking endogenous ATG4B expression. Therefore it is very important to distinguish between pro- and lipidated LC3B when assessing LC3B status under experimental conditions where ATG4 activity might be affected.

In conclusion, our study answers a fundamental question of mammalian autophagy in regards to the degree of genetic redundancy in the LC3–PE conjugation system. The discovery that ATG4B is the main human isoform but all other ATG4 isoforms broadly contribute to LC3/GABARAP processing in cells will be useful for designing autophagy inhibitors and predicting the effects of ATG4 perturbation *in vivo*. It will also provide a basis for understanding how post-translational modifications of ATG4 affect mammalian autophagy. Finally, our insights into human autophagy functions that do not require ATG4-mediated delipidation will inform future studies with the aim of specifically monitoring this elusive reaction in cells.

## Materials and methods

### Cell culture

HAP1 control (Horizon Genomics, C631) and HAP1 *ATG4B* KO cells (Horizon Genomics, HZGHC001241c011) were grown in IMDM containing 25 mM HEPES and L-Glutamine (ThermoFisher Scientific, 12440061) supplemented with Penicillin-Streptomycin (ThermoFisher Scientific, 15140122; 100 U/ml each) and 10% heat-inactivated FBS (ThermoFisher Scientific, 10500064). HeLa and HEK293T cells were grown in DMEM high glucose with GlutaMax and 1 mM pyruvate (ThermoFisher Scientific, 31966021), penicillin-streptomycin (100 U/ml) and 10% FBS (ThermoFisher Scientific, 10270106). All cells were maintained at 37°C, 5% CO_2_ and passaged at a dilution of 1:5–1:30 every 2–3 days. Cell line authentication performed by Eurofins was used to confirm the HeLa identity of main cell lines used in this study (HeLa control, *ATG4B* KO, *ATG4A/B* DKO c25, *ATG4A/B/C* TKO c22).

### Plasmid transfection

Quantities listed are per well for cells grown in 12-well plate format, and were scaled according to volume of growth medium used in other formats. HAP1 cells were transfected using Polyethyleneimine ‘MAX’ (Polysciences, 24765; stock dissolved at 1 mg/ml in sterile water and filter sterilized) at 3 µg per 1 µg of DNA, and 1 µg total DNA per 1 ml of growth medium. Mixtures of DNA and transfection reagent were diluted in 100 µl of IMDM without supplements, incubated for 15 min at room temperature, and then added directly to cells grown in complete IMDM. Cells were returned to incubator and medium was replaced after 4 h. HeLa cells were transfected with 250 ng DNA using 1.5 µl jetPRIME reagent (Polyplus-transfection, 114–15) and 50 µl jetPRIME buffer per 1 ml of growth medium. Experiments were performed at 24 h post-transfection.

### Generation of knockout cell lines using CRISPR-Cas9

Gene-specific sgRNA sequences for human *ATG4* isoforms were identified using Sanger CRISPR Finder tool (http://www.sanger.ac.uk/htgt/wge/find_crisprs) [35]. Sequences were chosen that targeted an early constitutive coding exon or residues close to the catalytic cysteine, and had the lowest possible probability of off-target cleavage in other gene regions. Oligonucleotides corresponding to desired guide sequences (listed in **Table S1**) were phosphorylated, annealed and cloned into BbsI-digested pSpCas9(BB)-2A-Puro (PX459) V2.0 (Addgene, 62988; Zhang lab) and delivered by transient transfection to generate knockout HeLa cell lines [36]. HeLa cells grown in 24-well plate format were transfected with 250–500 ng of DNA per well using PEI or jetPRIME. After 24 h, transfected cells were enriched by selection with 1 µg/ml puromycin for 2–3 days, following which puromycin was removed. Cells were then transferred to 10 cm dishes to recover for several days. For generation of HeLa *ATG4A/B* DKO and *ATG4A/B/C* TKO cells, the procedure was repeated 3 times in an attempt to increase the efficiency of gene disruption on multiple alleles. Single-cell clones were then isolated by limiting dilution in 96-well plates and grown for 2 weeks. Clones were screened for loss of target protein expression by western blot after expansion into 12-well plates. Positive clones were expanded and further validated by sequencing PCR-amplified genomic DNA of the target locus to confirm gene editing of all detected alleles. Loss of plasmid expression was demonstrated by confirming that clones had regained sensitivity to puromycin treatment. To generate HeLa control cells, unmodified HeLa cells were transfected with PX459 encoding a non-targeting sgRNA [37], selected with puromycin for 2 days, and then passaged as a pool for at least three weeks prior to use in experiments.

### Lentiviral production and stable cell line generation

HEK293T cells grown in a 6-well plate format were co-transfected with 900 ng psPAX2 (Addgene, 12260; Trono lab), 100 ng pMD2.G (Addgene, 12259; Trono lab) and 1 µg of transfer plasmid per well using X-tremeGENE HP (Roche, 6366244001) at 2 µl per µg of total DNA. At 18 h post-transfection, medium was replaced with complete IMDM. Viral supernatant was harvested at 48 and 72 h post-transfection, filtered through a 0.22-µm low protein-binding syringe filter and frozen at −80°C. Target cells seeded at a medium density in 24-well plates were infected overnight with 300 µl of filtered viral supernatant containing polybrene at a final concentration of 8 µg/ml. Cells were selected at 48 h post-infection with 1 µg/ml puromycin, expanded into 10-cm dishes and passaged once before puromycin was excluded from growth medium. Experiments were performed without puromycin present using pooled populations of transduced cells.

### RNA interference

HeLa cells grown in a 12-well format were transfected with a total of 500 ng MISSION esiRNA (Sigma) per well diluted in 100 µl jetPRIME buffer using 3 µl of jetPRIME reagent. This mixture was added directly to 1 ml of growth medium present in the well. In experiments where 2 targets were depleted simultaneously, the amount of esiRNA against each target was halved to keep the total amount of esiRNA constant. In these experiments, negative control esiRNA targeting *RLUC* was used to keep the esiRNA dosage constant in samples where only one target was depleted. Experiments were performed at 48 h after siRNA transfection. In siRNA experiments involving cDNA transfection, medium was changed at 24 h following siRNA transfection, and DNA transfection was subsequently performed as described. Catalog numbers and targets of esiRNAs are as follows: *RLUC* (*Renilla* luciferase; Sigma, EHURLUC), human *ATG4C* (Sigma, EHU060781), human *ATG4D* (Sigma, EHU035721), *EGFP* (enhanced GFP; Sigma, EHUEGFP), human *CASP3/caspase-3* (Sigma, EHU085021), human *ATG3* (Sigma, EHU015141), human *ATG7* (Sigma, EHU092581).

### Antibodies and reagents

Primary antibodies and dilutions used for western blot in this study were as follows: ATG3 (Abcam, ab108251; 1:1,000), ATG4A (Cell Signaling Technology, 7613; 1:1,000), ATG4B C terminus (Cell Signaling Technology, 5299; 1:1,000), ATG4B N terminus (Sigma, A2981; 1:1,000), ATG4C (Cell Signaling Technology, 5262; 1:200–1:500), ATG4D (Proteintech, 16924–1-AP; 1:1,000), ATG7 (Abcam, ab52472; 1:2,000), Beta-actin (Sigma, A1978; 1:4,000), CASP3/caspase-3 (Santa Cruz Biotechnology, sc-56053; 1:200), FLAG biotin-conjugated (Sigma, F9291; 1:1,000), GABARAP (Abgent, AP1821a; 1:1,000), GABARAPL1 (Proteintech, 11010–1-AP; 1:1,000), GABARAPL2 (Abcam, ab126607; 1:500), GFP (Clontech, 632381; 1:2,000), LAMP1 (BD Biosciences, 611043; 1:500), LC3A/B (Cell Signaling Technology, 12741; 1:500), LC3B (Sigma, L7542; 1:1,000 was used for LC3B western blotting unless otherwise stated) and (Cell Signaling Technology, 3868; 1:1,000 was used for western blotting in Figure S1B only), MFN2/mitofusin 2 (Abcam, ab56889; 1:500), SQSTM1/p62 (Sigma, P0067; 1:1,000), PDHA1/pyruvate dehydrogenase E1 alpha 1 subunit (Abcam, ab110330; 1:1,000), ubiquitin (Santa Cruz Biotechnology, sc-8017; 1:500), ULK1 (Cell Signaling Technology, 4733; 1:500), p-ULK1 S757/phospho-ULK1 Ser747 (Cell Signaling Technology, 6888; 1:1,000), VCL/vinculin (Abcam, ab129002; 1:1,000). Secondary antibodies used were: goat anti-mouse/rabbit IgG HRP (Cell Signaling Technology, 7076/7074; 1:5,000), IRDye 800CW goat anti-rabbit (LI-COR Biosciences, 926–32211; 1:15,000 with 0.02% SDS) and IRDye 680LT goat anti-mouse (LI-COR Biosciences, 926–68020; 1:25,000 with 0.02% SDS).

For immunocytochemistry, the following antibodies and dilutions were used: CANX/calnexin (BD Biosciences, 610523; 1:50), EEA1 (BD Biosciences, 610456; 1:200), GABARAPL1 (Proteintech, 11010–1-AP; 1:200), GFP (Abcam, ab290; 1:500), GOLGA2/GM130 (BD Biosciences, 610822; 1:500), LAMP1 (BD Biosciences, 555798; 1:1,000), LC3B (Cell Signaling Technology, 3868; 1:200), SQSTM1/p62 (Sigma, P0067; 1:1,000). Secondary fluorescent antibodies used were: Alexa Fluor 488/568 goat anti-rabbit IgG (H + L; ThermoFisher Scientific, A-11008/11011; 1:400), Alexa Fluor 647 goat anti-mouse IgG (H + L; ThermoFisher Scientific, A-21235; 1:400).

Torin1 (Merck-Millipore, 475991) was dissolved in DMSO at a final stock concentration of 250 µM. Bafilomycin A_1_ from *Streptomyces griseus* (Sigma, B1793) was dissolved in DMSO at a final stock concentration of 10 µM. Recombinant GST-ATG4B WT was described previously [38]. Other reagents were as follows: PLD/phospholipase D from *Streptomyces chromofuscus* (Sigma, P0065), EBSS containing calcium and magnesium (ThermoFisher Scientific, 24010–043). For the protease protection assay, Trypsin from porcine pancreas was used (Sigma, T4799).

### Plasmids and cloning

Expression constructs of tagged WT and mutant human LC3/GABARAP isoforms were generated using Gateway cloning technology (Life Technologies). Entry clones were first generated by PCR using complementary or mutagenic primers with attB sequence overhangs (listed in **Table S2**) followed by recombination with pDONR223 using BP clonase II enzyme mix (ThermoFisher Scientific, 11789020) [39]. To generate N-terminal 3xFLAG-tagged constructs, entry clones containing stop codons were recombined with the destination vector pCMV-tripleFLAG-Gateway [40] using LR clonase II enzyme mix (ThermoFisher Scientific, 11791020). To make transient expression constructs of GFP-LC3/GABARAP, pDEST-CMV-N-EGFP was used as the destination vector. For generating 3xFLAG-ATG8-EGFP double-tagged constructs, entry clones lacking stop codons were recombined with pDEST-CMV-3xFLAG-gateway-EGFP. To generate lentiviral transfer vectors for the production of cell lines stably expressing *CMV* promoter-driven GFP-tagged ATG8 isoforms, entry clones containing stop codons were recombined with pLVpuro-CMV-N-EGFP.

Novel destination vector constructs were generated as follows. EGFP was PCR amplified from pEAK13-EGFP using the primers 5ʹ-GFP-Kpn1 (5ʹ-acgtaGGTACCGCCACCATGGTGAGCAAGGGCGAGGAGCTGTTC-3ʹ) and 3ʹ-GFP-HindIII (5ʹ-acgtaAAGCTTCTTGTACAGCTCGTCCATGCCGAGAGTGATC-3ʹ), cut and ligated into pCMV-tripleFLAG-Gateway using KpnI and HindIII enzymes to generate pCMV-EGFP-gateway. A portion of the *CMV* promoter and *EGFP* was then PCR amplified using the primers 5ʹ-CMV-SacI (5ʹ-gacGAGCTCGTTTAGTGAACCGTC-3ʹ) and 3ʹ-EGFP-SacI (5ʹ-gacGAGCTCGTCCATGCCGTGAGTGATC-3ʹ), cut and ligated into Gateway® pcDNA™-DEST53 (ThermoFisher Scientific, 12288015) using SacI digestion to generate pDEST-CMV-N-EGFP. The EGFP tag and gateway cassette portion of pDEST-CMV-N-EGFP was PCR amplified using the primers 5ʹ-EGFP-NheIPacIXbaI (5ʹ-GACGCTAGCTTAATTAAGACTCTAGAGCCACCATGGTGAGCAAG-3ʹ) and 3ʹ-attR2-BstBI (5ʹ-gacTTCGAATCACACCACTTTGTACAAGAAAG-3ʹ) and cloned into pLV-CMV-y/hNubI-tripleFLAG-linker-Gateway- PuroR using NheI and BstBI digestion to generate pLVpuro-CMV-N-EGFP. The gateway cassette and 3ʹ linker region of Gateway® pcDNA™-DEST47 (ThermoFisher Scientific, 12281010) was PCR amplified using the primers 5ʹ-gateway-KpnIHpaINheI (5ʹ-GACGGTACCGGCGTTAACGACGCTAGCGGagttaagcttgatcaaacaagtttg-3ʹ) and 3ʹ-linker-XhoIPmeIPacI (5ʹ-GACTTAATTAAGCCGGTTTAAACACCGCTCGAGtctagatcgaaccactttgtaca-3ʹ) and ligated into pDEST-CMV-N-EGFP following digestion with KpnI and PacI to generate pDEST-CMV-polysite. EGFP was PCR amplified from pEAK13-EGFP using the primers 5ʹ-EGFP-XhoI (5ʹ-GACCTCGAGATGGTGAGCAAGGGCGAG-3ʹ) and 3ʹ-EGFP-stop-PacI (5ʹ-GACTTAATTAATTACTTGTACAGCTCGTCCATG-3ʹ) and ligated into pDEST-CMV-polysite after digestion with XhoI and PacI to generate pDEST-CMV-C-EGFP. 3xFLAG-attR1 fragment was digested from pCMV-tripleFLAG-Gateway and subcloned into pDEST-CMV-C-EGFP following digestion with KpnI and NotI to generate pDEST-CMV-3xFLAG-gateway-EGFP.

To generate lentiviral transfer plasmids for the production of stable cell lines expressing *PGK* promoter-driven LC3B or GABARAPL1 mutants, the GFP-LC3/GABARAP coding sequence was PCR amplified from the respective variant of pDEST-CMV-N-EGFP and subcloned by restriction digests between the BamHI and SalI sites of pLenti PGK GFP Puro (w509-5) (Addgene, 19070; Campeau and Kaufman labs) [41]. These PCRs were performed using the common forward primer 5ʹ-EGFP-BamHI (5ʹ- GACGGATCCGCCACCATGGTGAGCAAG-3ʹ) and one of the following reverse primers: 3ʹ-LC3B-G120-SalI (5ʹ- GACgtcgacTTACCCGAACGTCTCCTGG-3ʹ), 3ʹ-LC3B-GA-SalI (5ʹ-GACgtcgacTTACACTGACAATTTCATCGCG-3ʹ), 3ʹ-GABARAPL1-G116-SalI (5ʹ-GACgtcgacTTACCCATAGACACTCTCATCA-3ʹ) or 3ʹ-GABARAPL1-GA-SalI (5ʹ-GACgtcgacTTATTTCGCATAGACACTCTCATC-3ʹ). All cloning was confirmed by diagnostic restriction enzyme digests and Sanger sequencing of coding sequences.

### Genomic DNA isolation and analysis

Genomic DNA was isolated using NucleoSpin Tissue genomic DNA purification kit (Macherey-Nagel, 740952) according to the manufacturer’s protocol for cultured cells. The genomic locus of interest was PCR-amplified using specific primers (listed in **Table S1**) designed using NCBI Primer-BLAST (https://www.ncbi.nlm.nih.gov/tools/primer-blast) [42] against relevant regions of reference genome sequence retrieved using the ‘Table Browser’ function of UCSC Genome Browser (https://genome.ucsc.edu/cgi-bin/hgTables) [43]. Genomic primers incorporated attB sequence overhangs to allow subsequent gateway cloning of gel-purified PCR product into pDONR223 using BP Clonase II enzyme mix. The reaction mix was transformed into bacteria and plasmid DNA was extracted from overnight cultures of multiple individual clones and sequenced by Sanger sequencing using M13 F and R primers. Genotyping results of main CRISPR-modified clones used in this study are provided in **Table S3.**

### Western blotting

Cells were washed in ice-cold PBS (137 mM NaCl, 2.7 mM KCl, 10 mM Na_2_HPO_4_, 1.8 mM KH_2_PO_4_, pH 7.4) prior to lysis in NP-40 lysis buffer (150 mM NaCl, 1% IGEPAL® CA-630/NP-40 substitute [Sigma, I8896], 50 mM Tris-HCl, pH 8.0) containing protease inhibitors (c0mplete, EDTA-free, Roche, 11873580001) and 20 mM N-ethylmaleimide (unless otherwise stated) on ice. Lysates were cleared by centrifugation at 15,200 x g at 4°C and the resulting pellet was discarded. Protein levels were quantified using Pierce BCA Protein Assay Kit (ThermoFisher Scientific, 23,225) and lysates were diluted to approximately equal concentrations before heating in SDS sample buffer (with final concentration in the sample of 50 mM Tris-Cl, pH 6.8, 2% SDS, 10% glycerol, 5% beta-mercaptoethanol, 0.01% bromophenol blue) at 95°C for 5 min. Equal amounts of sample (typically 25 µg total protein per lane) were loaded into lanes of a 15-well 4–20% Mini-PROTEAN TGX Precast Gel or 26-well 4–20% Criterion TGX gel (Bio-Rad) and run at 115 V constant for 75 min or 200 V for 35 min. Protein was transferred to Immobilon-FL PVDF membrane (Merck-Millipore, IPFL00010) before blocking with 5% skimmed milk in PBS-T (PBS containing 0.05% Tween® 20 [Sigma, P1379]). The membrane was probed with primary antibody in blocking buffer at room temperature for 1 h or overnight at 4°C. After washing with PBS-T, the membrane was incubated in secondary antibody in blocking buffer for 1 h at room temperature. Further washes were carried out before image acquisition on an Odyssey infrared imaging system (LI-COR Biosciences) or development using EZ-ECL chemiluminescence detection kit for HRP (Geneflow Ltd, K1-0172) followed by exposure on an ImageQuant chemilumenscent imaging system (GE Healthcare).

### PLD bandshift assay

After treatment with compounds, cells were lysed on ice in PLD assay buffer (150 mM NaCl, 50 mM Tris-HCl, pH 8.0, 5 mM CaCl_2_, 1% Triton X-100 [Sigma, X100]) without EDTA or protease inhibitors, because PLD requires Ca^2+^ for its activity [44]. Lysates were cleared by centrifugation and then divided into separate tubes of equal volumes. On ice, PLD was added to a final concentration of 2.5 units/µl, or GST-ATG4B was added to a final concentration of 0.1 µg/µl. For negative control samples, an equivalent volume of PLD assay buffer was added. Samples were then heated to 37°C for 1 h while an unheated sample was kept on ice for this duration as a control. The reaction was stopped by the addition of 5X SDS sample buffer (250 mM Tris-Cl, pH 6.8, 10% SDS, 50% glycerol, 25% beta-mercaptoethanol, 0.05% bromophenol blue) to the tube with immediate boiling at 95°C for 5 min. Equal volumes of sample were then loaded on a 4–20% gel and assessed by western blot.

### Protease-protection assay

Our protease-protection assay was adapted and optimized from a combination of 2 published protocols [45,46]. Cells were seeded 24 h prior to transfection in one 10-cm dish per sample. The day after transfection, cells were treated with Torin1 and baf A1 for 3 h before harvesting by trypsinization (ThermoFisher Scientific, 25200056) at 37°C. Trypsin for harvesting was inactivated by resuspending intact cells in pre-warmed complete growth medium. Cells were then gently centrifuged and washed once in ice-cold PBS to remove traces of trypsin. Cells were again centrifuged and resuspended in 0.7 ml of ice-cold homogenization buffer (HB: 10 mM HEPES-KOH, pH 7.5, 0.22 M D-mannitol, 0.07 M sucrose [Sigma, S0389], 1 mM EDTA, 1 mM DTT). All subsequent steps were performed on ice or at 4°C. Cells were ruptured by passing the suspension 10 times through a 27G needle attached to a 1 ml syringe. Following this, cells were centrifuged twice at 300 x g and the nuclear pellet was discarded. Each sample was then divided into 2 tubes and permeabilized or not permeabilized by addition of 5% Triton X-100 in HB to a final concentration of 0.5%, or an equivalent volume of HB. These mixtures were then further subdivided into 3 tubes, one of which had 1 mg/ml trypsin (dissolved fresh in HB) added to a final concentration of 100 µg/ml, while the other 2 untreated tubes had an equivalent volume of HB added. Two were then incubated at 37°C for 30 min, while one untreated tube was kept on ice as a control. To stop the reactions, all tubes were placed on ice and 5X SDS sample buffer was added, before immediate boiling at 95°C for 10 min. Equal volumes of sample were then immediately loaded on 4–20% polyacrylamide gels and analyzed by western blotting.

### Immunocytochemistry and light microscopy

Cells seeded on borosilicate glass coverslips (13-mm diameter, thickness no. 1.5; VWR, 631–0150) were washed in PBS and fixed for 12 min at room temperature in 4% paraformaldehyde in PBS. For imaging fluorescent proteins alone, the coverslips were then washed, stained with 1 µg/ml Hoechst 33342 (ThermoFisher Scientific, H3570) for 10 min, washed and mounted onto glass slides using ProLong Diamond mounting medium (ThermoFisher Scientific, P36970) and cured overnight at room temperature in the dark. For immunocytochemistry, cells were permeabilized and fixed a second time in ice cold methanol at −20°C for 12 min, washed in PBS, quenched in 50 mM NH_4_Cl in PBS for 20 min, washed, and incubated in blocking buffer (3% goat serum [ThermoFisher Scientific, 16210064] in PBS) for 1 h. Coverslips were transferred to humidified staining boxes and in incubated overnight at 4°C with 45 µl primary antibody diluted in blocking buffer. Coverslips were then washed 3 times with PBS for a total of 10 min, and then incubated with 45 µl secondary antibody in blocking buffer containing 1 µg/ml Hoechst 33342 for 1 h at room temperature, prior to washing and mounting onto slides. For co-staining experiments, antibodies raised in different species against different target proteins were incubated simultaneously. Fixed cells were imaged on a Leica SPE, inverted SPE, SP5 or SP8 confocal laser-scanning microscope using a 63x oil immersion objective with a numerical aperture (N.A.) of 1.3 (SPE, inverted SPE) or 1.4 (SP5, SP8) at 400 Hz with sequential channel acquisition. Laser and gain settings were optimized for the brightest sample to avoid signal saturation, and the same settings were used to image all samples within a single experiment. All images shown are of a single confocal Z-slice. For some confocal microscopy figures, the contrast level was increased in a linear manner using the ‘Levels’ tool in Adobe Photoshop to improve clarity. When performed, this adjustment was applied equally and uniformly to all experimental conditions and controls.

### Transmission electron microscopy

Cells seeded on glass coverslips were first fixed by addition of an equal volume of double-concentrate fixative solution (3% formaldehyde, 0.2% glutaraldehyde in PBS) for 15 min at room temperature. Cells were then fixed a second time in 2% glutaraldehyde in 0.1 M sodium cacodylate for 15 min. Coverslips were then rinsed twice in 0.1 M sodium cacodylate and fixed in 1% osmium tetroxide 1.5% potassium ferrocyanide for 1 h at 4°C. After rinsing 3 times in 0.1 M sodium cacodylate, samples were stained with 1% (w:v) tannic acid in 0.05 M sodium cacodylate for 45 min at room temperature in the dark, before incubation with 1% (w:v) sodium sulfate in 0.05 M sodium cacodylate. Samples were then dehydrated by sequential 5-min incubations in distilled water, followed by 70% ethanol (x2), 90% ethanol (x2) and 100% dry ethanol (2x). Coverslips were then transferred to a 1:1 (v:v) mix of propylene oxide:TAAB 812 resin (TAAB Laboratories Equipment Ltd, T024) for 1 h. Samples were transferred again twice to fresh resin for 1.5 h each time. Coverslips were then mounted onto pre-polymerized blocks with a drop of fresh resin and baked overnight at 60°C. Glass was removed by cooling in liquid nitrogen and 70-nm ultrathin sections were cut parallel to the growth surface using a diamond knife and collected on formvar-coated copper slot grids (Agar Scientific, AGG2525C). Sections were stained with lead citrate, washed 6 times with distilled water, and air dried before imaging on a Tecnai Spirit (FEI) with a Morada digital camera (EMSIS) at 120 kV.

### Correlative light and electron microscopy

Stably transduced cells were seeded on a gridded glass coverslip in a 35-mm cell culture dish (MatTek, P35G-2–14-C-GRID). The next day, cells were treated with compounds, and MitoTracker Red cmxROS (Lonza, PA-3017) was added to the growth medium at a concentration of 100 nM for the final 45 min of the experiment. Cells were fixed by addition of an equal volume of double-concentrate fixative solution (3% formaldehyde, 0.2% glutaraldehyde, 2 µg/ml Hoechst 33342 in PBS) for 15 min at room temperature. Cells were then washed twice in PBS and left in PBS for imaging. A cell near the center of the coverslip was imaged on a Leica inverted SP5 confocal laser scanning microscope with DIC using a 63x numerical aperture 1.4 oil immersion objective. Z-stacks were acquired at a slice separation of 300 nm and channels were acquired sequentially for each slice in turn. A DIC image showing the morphology of the cell and its grid position was acquired using a 40x objective. After imaging, samples were fixed a second time with 2% glutaraldehyde 0.1 M sodium cacodylate for 15 min at room temperature. After washing, samples were osmium fixed, stained, dehydrated and embedded as described above for conventional TEM. The same cell imaged by confocal microscopy was then re-located on the block face by referring to its grid position and morphology from the DIC image. The block was trimmed to this region and serial sections were collected, stained, and imaged by TEM as above. Light microscopy data were adjusted for clarity in a linear manner using brightness and contrast tools, prior to being manually aligned with electron microscopy data using the transform and layers tools in Adobe Photoshop. DIC images and TEM low power overview, together with MitoTracker signal and mitochondria ultrastructure, were used as unbiased fiducial markers.

### Morphometric analysis of autophagosomes from TEM images

Olympus iTEM software was used. For each cell, cytoplasm area was first calculated using a low magnification image by outlining the entire cell using the interpolated polygon tool, measuring total area, and subtracting the nuclear area. Autophagosomes were then identified, using higher magnification images, based on their distinctive morphology of cytoplasmic contents within membrane-limited compartments. To measure autophagosome size, the outer limiting membrane of each structure was traced using the freehand selection tool in 2D.

### Densitometry

Protein levels were quantified using the Analyze>Gel function in FIJI/ImageJ (NIH). The raw unprocessed western blot image was opened and a box was drawn surrounding the band of interest. After background subtraction, the histogram area of the signal of each band was determined and divided by the corresponding signal of a loading control band (ACTIN or VCL) from the same gel lane.

### Image analysis

Puncta of endogenous LC3B and GABARAPL1 were counted in an unbiased semi-automated manner using the ‘Find Maxima’ tool in FIJI/ImageJ (NIH), with a noise tolerance value of 150 or 140, respectively. The nuclear region was first excluded from analysis by inverting the selection of a binary image generated using the Hoechst channel. Automatically identified puncta were manually counted and assigned to a parent cell to generate each data point. Colocalization between SQSTM1 and LAMP1 was assessed using the Coloc 2 plugin in FIJI. An individual cell was manually selected as an ROI using the polygon selection tool to generate each data point.

### Statistical analysis

All statistical tests were done using GraphPad Prism. Unpaired two-tailed t-tests were performed for experiments comparing two sets of data. For experiments involving the comparison of multiple sets of data, an ordinary one-way ANOVA was performed using Sidak’s multiple comparison post-test to compare relevant pre-defined dataset pairs.

## References

[1] Bento CF, Renna M, Ghislat G, et al Mammalian autophagy: how does it work? Annu. Rev Biochem. 2016;85:685–713. 10.1146/annurev-biochem-060815-014556 26865532

[2] Galluzzi L, Baehrecke EH, Ballabio A, et al Molecular definitions of autophagy and related processes. Embo J. 2017;36:1811–1836. 28596378 10.15252/embj.201796697 PMC5494474

[3] Harding TM, Morano KA, Scott SV, et al Isolation and characterization of yeast mutants in the cytoplasm to vacuole protein targeting pathway. J Cell Biol. 1995;131:591–602. 7593182 10.1083/jcb.131.3.591 PMC2120622

[4] Tsukada M, Ohsumi Y. Isolation and characterization of autophagy-defective mutants of Saccharomyces cerevisiae. FEBS Lett. 1993;333:169–174. 8224160 10.1016/0014-5793(93)80398-e

[5] Ichimura Y, Kirisako T, Takao T, et al A ubiquitin-like system mediates protein lipidation. Nature. 2000;408:488–492. 11100732 10.1038/35044114

[6] Hanada T, Noda NN, Satomi Y, et al The Atg12-Atg5 conjugate has a novel E3-like activity for protein lipidation in autophagy. J Biol Chem. 2007;282:37298–37302. 17986448 10.1074/jbc.C700195200

[7] Xie Z, Nair U, Klionsky DJ Atg8 controls phagophore expansion during autophagosome formation. Mol Biol Cell. 2008;19:3290–3298. 18508918 10.1091/mbc.E07-12-1292 PMC2488302

[8] Pankiv S, Clausen TH, Lamark T, et al p62/SQSTM1 binds directly to Atg8/LC3 to facilitate degradation of ubiquitinated protein aggregates by autophagy. J Biol Chem. 2007;282:24131–24145. 17580304 10.1074/jbc.M702824200

[9] Kirisako T, Ichimura Y, Okada H, et al The reversible modification regulates the membrane-binding state of Apg8/Aut7 essential for autophagy and the cytoplasm to vacuole targeting pathway. J Cell Biol. 2000;151:263–276. 11038174 10.1083/jcb.151.2.263 PMC2192639

[10] Nair U, Yen W-L, Mari M, et al A role for Atg8-PE deconjugation in autophagosome biogenesis. Autophagy. 2012;8:780–793. 22622160 10.4161/auto.19385 PMC3378420

[11] Nakatogawa H, Ishii J, Asai E, et al Atg4 recycles inappropriately lipidated Atg8 to promote autophagosome biogenesis. Autophagy. 2012;8:177–186. 22240591 10.4161/auto.8.2.18373

[12] Yu Z-Q, Ni T, Hong B, et al Dual roles of Atg8-PE deconjugation by Atg4 in autophagy. Autophagy. 2012;8:883–892. 22652539 10.4161/auto.19652 PMC3427254

[13] Tsuboyama K, Koyama-Honda I, Sakamaki Y, et al The ATG conjugation systems are important for degradation of the inner autophagosomal membrane. Science. 2016;354:1036–1041. 27885029 10.1126/science.aaf6136

[14] Nguyen TN, Padman BS, Usher J, et al Atg8 family LC3/GABARAP proteins are crucial for autophagosome-lysosome fusion but not autophagosome formation during PINK1/Parkin mitophagy and starvation. J Cell Biol. 2016;215:857–874. 27864321 10.1083/jcb.201607039 PMC5166504

[15] Weidberg H, Shvets E, Shpilka T, et al LC3 and GATE-16/GABARAP subfamilies are both essential yet act differently in autophagosome biogenesis. Embo J. 2010;29:1792–1802. 20418806 10.1038/emboj.2010.74 PMC2885923

[16] Li M, Hou Y, Wang J, et al Kinetics comparisons of mammalian Atg4 homologues indicate selective preferences toward diverse Atg8 substrates. J Biol Chem. 2011;286:7327–7338. 21177865 10.1074/jbc.M110.199059 PMC3044989

[17] Scherz-Shouval R, Sagiv Y, Shorer H, et al The COOH terminus of GATE-16, an intra-Golgi transport modulator, is cleaved by the human cysteine protease HsApg4A. J Biol Chem. 2003;278:14053–14058. 12473658 10.1074/jbc.M212108200

[18] Betin VMS, Lane JD Caspase cleavage of Atg4D stimulates GABARAP-L1 processing and triggers mitochondrial targeting and apoptosis. J Cell Sci. 2009;122:2554–2566. 19549685 10.1242/jcs.046250 PMC2704886

[19] Mariño G, Fernández AF, Cabrera S, et al Autophagy is essential for mouse sense of balance. J Clin Invest. 2010;120:2331–2344. 20577052 10.1172/JCI42601 PMC2898610

[20] Sou Y, Waguri S, Iwata J, et al The Atg8 conjugation system is indispensable for proper development of autophagic isolation membranes in mice. Mol Biol Cell. 2008;19:4762–4775. 18768753 10.1091/mbc.E08-03-0309 PMC2575156

[21] Pengo N, Agrotis A, Prak K, et al A reversible phospho-switch mediated by ULK1 regulates the activity of autophagy protease ATG4B. Nat Commun. 2017;8:294. 28821708 10.1038/s41467-017-00303-2 PMC5562857

[22] Kim J, Kundu M, Viollet B, et al AMPK and mTOR regulate autophagy through direct phosphorylation of Ulk1. Nat Cell Biol. 2011;13:132–141. 21258367 10.1038/ncb2152 PMC3987946

[23] Wang W, Chen Z, Billiar TR, et al The carboxyl-terminal amino acids render pro-human LC3B migration similar to lipidated LC3B in SDS-PAGE. PloS One. 2013;8:e74222. 24040206 10.1371/journal.pone.0074222 PMC3769297

[24] Tanida I, Sou Y, Ezaki J, et al HsAtg4B/HsApg4B/autophagin-1 cleaves the carboxyl termini of three human Atg8 homologues and delipidates microtubule-associated protein light chain 3- and GABAA receptor-associated protein-phospholipid conjugates. J Biol Chem. 2004;279:36268–36276. 15187094 10.1074/jbc.M401461200

[25] Skytte Rasmussen M, Mouilleron S, Kumar Shrestha B, et al ATG4B contains a C-terminal LIR motif important for binding and efficient cleavage of mammalian orthologs of yeast Atg8. Autophagy. 2017;13:834–853. 28287329 10.1080/15548627.2017.1287651 PMC5446077

[26] Le Grand JN, Chakrama FZ, Seguin-Py S, et al GABARAPL1 antibodies: target one protein, get one free! Autophagy. 2011;7:1302–1307. 21862879 10.4161/auto.7.11.16723

[27] Ylä-Anttila P, Vihinen H, Jokitalo E, et al Monitoring autophagy by electron microscopy in mammalian cells. Methods Enzymol. 2009;452:143–164. 19200881 10.1016/S0076-6879(08)03610-0

[28] Bjørkøy G, Lamark T, Brech A, et al p62/SQSTM1 forms protein aggregates degraded by autophagy and has a protective effect on huntingtin-induced cell death. J Cell Biol. 2005;171:603–614. 16286508 10.1083/jcb.200507002 PMC2171557

[29] Kauffman KJ, Yu S, Jin J, et al Delipidation of mammalian Atg8-family proteins by each of the four ATG4 proteases. Autophagy. 2018;1–56. 10.1080/15548627.2018.1437341 PMC6103404 29458288

[30] Kabeya Y, Mizushima N, Ueno T, et al LC3, a mammalian homologue of yeast Apg8p, is localized in autophagosome membranes after processing. Embo J. 2000;19:5720–5728. 11060023 10.1093/emboj/19.21.5720 PMC305793

[31] Kimura S, Noda T, Yoshimori T Dissection of the autophagosome maturation process by a novel reporter protein, tandem fluorescent-tagged LC3. Autophagy. 2007;3:452–460. 17534139 10.4161/auto.4451

[32] Fujita N, Hayashi-Nishino M, Fukumoto H, et al An Atg4B mutant hampers the lipidation of LC3 paralogues and causes defects in autophagosome closure. Mol Biol Cell. 2008;19:4651–4659. 18768752 10.1091/mbc.E08-03-0312 PMC2575160

[33] Abreu S, Kriegenburg F, Gómez-Sánchez R, et al Conserved Atg8 recognition sites mediate Atg4 association with autophagosomal membranes and Atg8 deconjugation. EMBO Rep. 2017;18:765–780. 28330855 10.15252/embr.201643146 PMC5412903

[34] Sánchez-Wandelmer J, Kriegenburg F, Rohringer S, et al Atg4 proteolytic activity can be inhibited by Atg1 phosphorylation. Nat Commun. 2017;8:295. 28821724 10.1038/s41467-017-00302-3 PMC5562703

[35] Hodgkins A, Farne A, Perera S, et al WGE: a CRISPR database for genome engineering. Bioinforma Oxf Engl. 2015;31:3078–3080. 10.1093/bioinformatics/btv308 PMC4565030 25979474

[36] Ran FA, Hsu PD, Wright J, et al Genome engineering using the CRISPR-Cas9 system. Nat Protoc. 2013;8:2281–2308. 24157548 10.1038/nprot.2013.143 PMC3969860

[37] Sanjana NE, Shalem O, Zhang F Improved vectors and genome-wide libraries for CRISPR screening. Nat Methods. 2014;11:783–784. 25075903 10.1038/nmeth.3047 PMC4486245

[38] Costa JR, Prak K, Aldous S, et al Autophagy gene expression profiling identifies a defective microtubule-associated protein light chain 3A mutant in cancer. Oncotarget. 2016;7:41203–41216. 27256984 10.18632/oncotarget.9754 PMC5173052

[39] Rual J-F, Hirozane-Kishikawa T, Hao T, et al Human ORFeome version 1.1: a platform for reverse proteomics. Genome Res. 2004;14:2128–2135. 15489335 10.1101/gr.2973604 PMC528929

[40] Petschnigg J, Groisman B, Kotlyar M, et al The mammalian-membrane two-hybrid assay (MaMTH) for probing membrane-protein interactions in human cells. Nat Methods. 2014;11:585–592. 24658140 10.1038/nmeth.2895

[41] Campeau E, Ruhl VE, Rodier F, et al A versatile viral system for expression and depletion of proteins in mammalian cells. PloS One. 2009;4:e6529. 19657394 10.1371/journal.pone.0006529 PMC2717805

[42] Ye J, Coulouris G, Zaretskaya I, et al Primer-BLAST: a tool to design target-specific primers for polymerase chain reaction. BMC Bioinformatics. 2012;13:134. 22708584 10.1186/1471-2105-13-134 PMC3412702

[43] Karolchik D, Hinrichs AS, Furey TS, et al The UCSC table browser data retrieval tool. Nucleic Acids Res. 2004;32:D493–496. 14681465 10.1093/nar/gkh103 PMC308837

[44] Yang H, Roberts MF Cloning, overexpression, and characterization of a bacterial Ca2+-dependent phospholipase D. Protein Sci Publ Protein Soc. 2002;11:2958–2968. 10.1110/ps.0225302 PMC2373738 12441393

[45] Diao J, Liu R, Rong Y, et al ATG14 promotes membrane tethering and fusion of autophagosomes to endolysosomes. Nature. 2015;520:563–566. 25686604 10.1038/nature14147 PMC4442024

[46] Velikkakath AKG, Nishimura T, Oita E, et al Mammalian Atg2 proteins are essential for autophagosome formation and important for regulation of size and distribution of lipid droplets. Mol Biol Cell. 2012;23:896–909. 22219374 10.1091/mbc.E11-09-0785 PMC3290647

