# Supplementary material for: Redundancy of human ATG4 protease isoforms in autophagy and LC3/GABARAP processing revealed in cells
Source: Autophagy. 2019 Feb 1;15(6):976–97. doi: 10.1080/15548627.2019.1569925 (PMC6526816; doi:10.1080/15548627.2019.1569925)
Supplement: Supplemental Material [file kaup-15-06-1569925-s0001.docx]

SUPPLEMENTARY FIGURES, TABLES AND LEGENDS


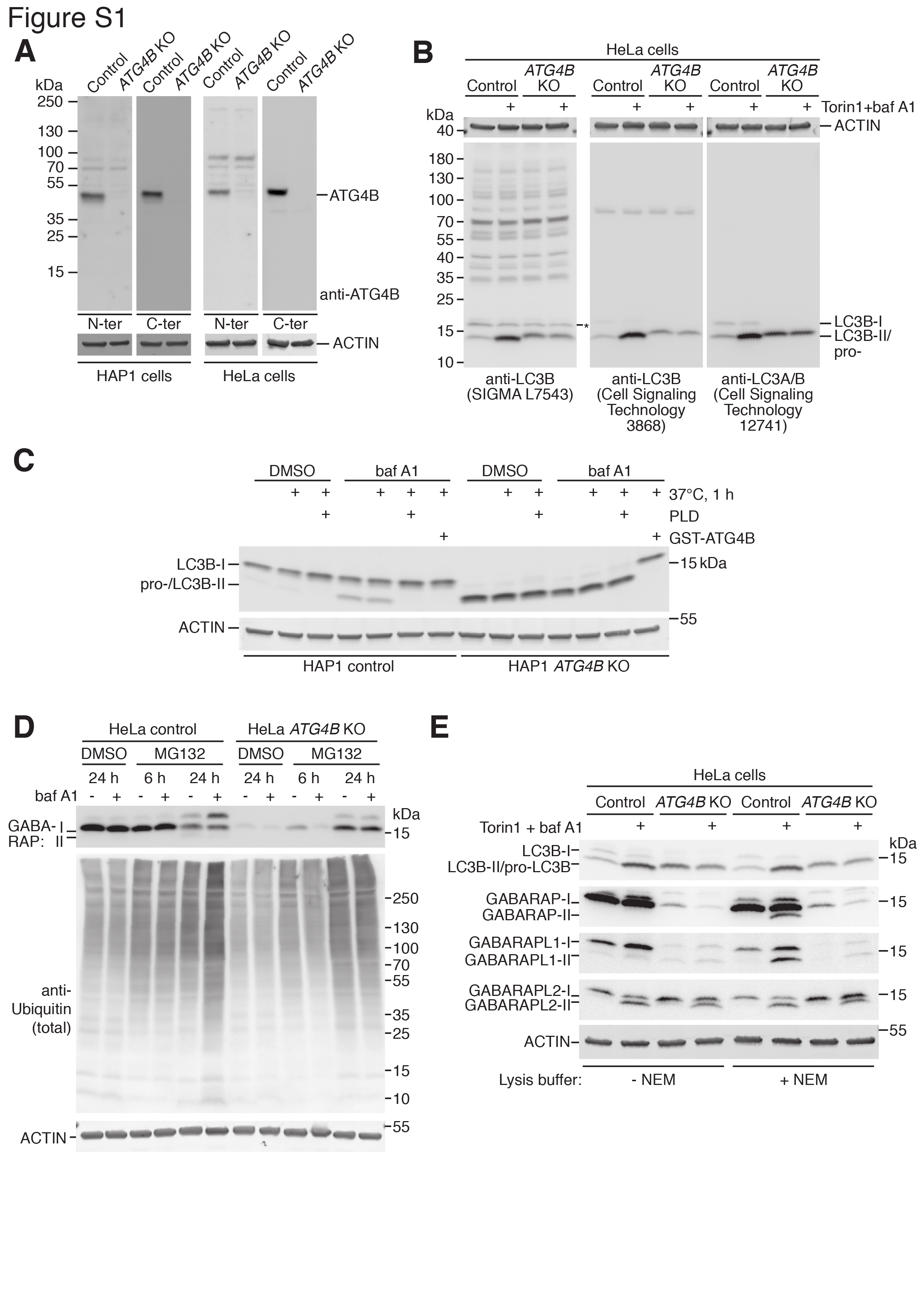
**Figure S1.** Additional characterization of human *ATG4B* KO cells. (**A**) Protein-level validation of CRISPR-Cas9-mediated *ATG4B* knockout. Lysates from control and *ATG4B* KO HAP1 and HeLa cells were subject to western blotting using polyclonal ATG4B antibodies raised against the N terminus (SIGMA, A2981) and C terminus (Cell Signaling Technology, 5299) of the protein. (**B**) Determination of background bands detected by anti-LC3B (SIGMA, L7543). HeLa control and *ATG4B* KO cells were treated for 3 h with DMSO or 250 nM Torin1 + baf A1 prior to lysis. Samples of the same lysates were run in triplicate on an SDS-PAGE gel before transfer to membrane. The membrane was cut and incubated with 3 different anti-LC3 antibodies as indicated. Asterisk indicates background band detected at the same molecular weight as LC3B-I. (**C**) PLD band shift assay performed on HAP1 control or *ATG4B* KO cells treated for 6 h with DMSO or 10 nM baf A1 and lysed in PLD assay buffer lacking NEM. Lysates were subject to *in vitro* treatment with purified PLD or GST-ATG4B prior to western blotting. (**D**) Western blot of lysates from HeLa control and *ATG4B* KO cells treated with the proteasome inhibitor MG132 (10 µM) for 6 or 24 h, in the presence or absence of 10 nM baf A1 added for the final 3 h of treatment. (**E**) Western blotting of LC3/GABARAP proteins using lysates from HeLa control and *ATG4B* KO cells treated for 3 h with DMSO or 250 nM Torin1 and 10 nM baf A1. Cells were lysed in the presence or absence of 20 mM NEM.


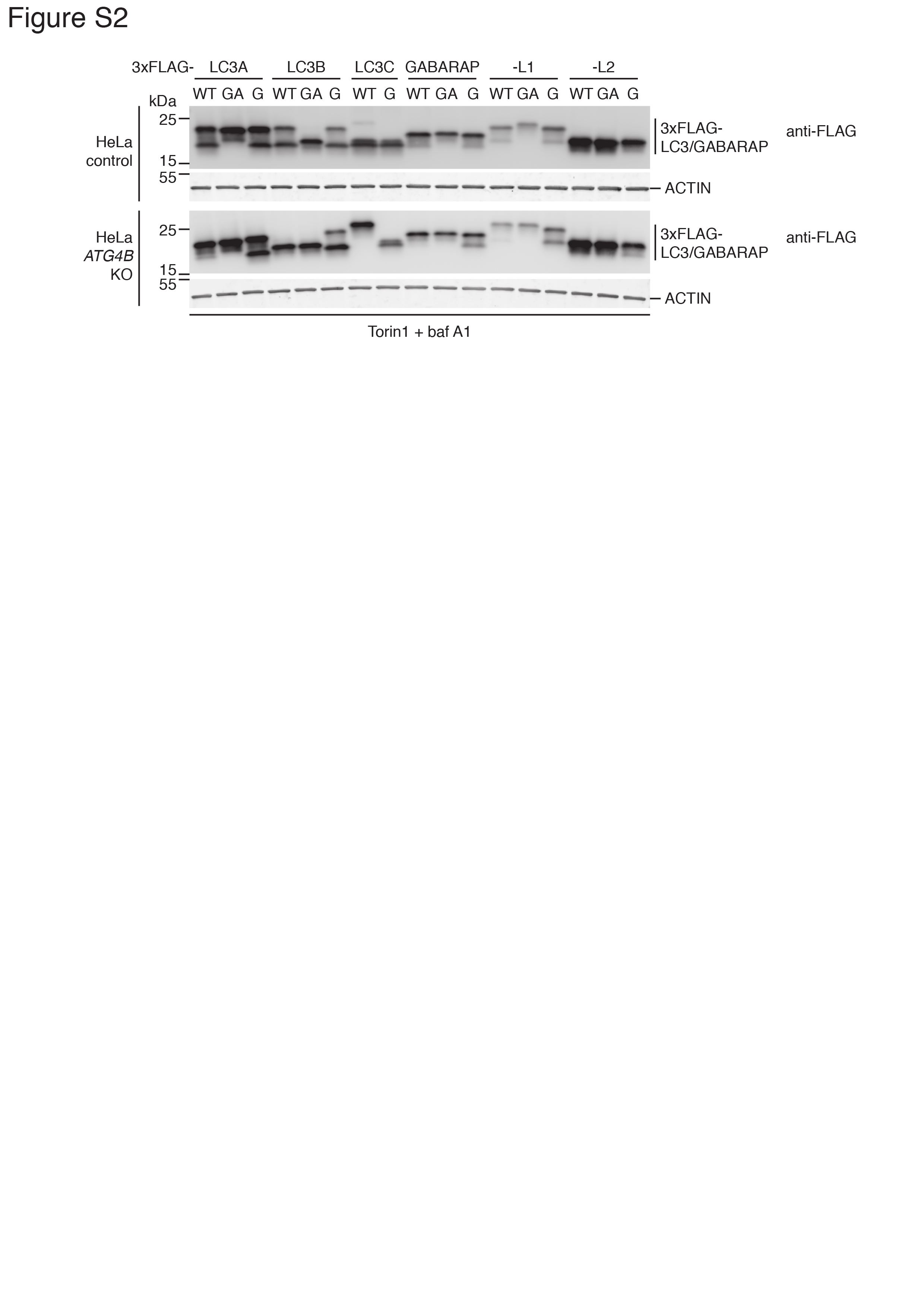


**Figure S2.** Original blots for Figure 2D**.**


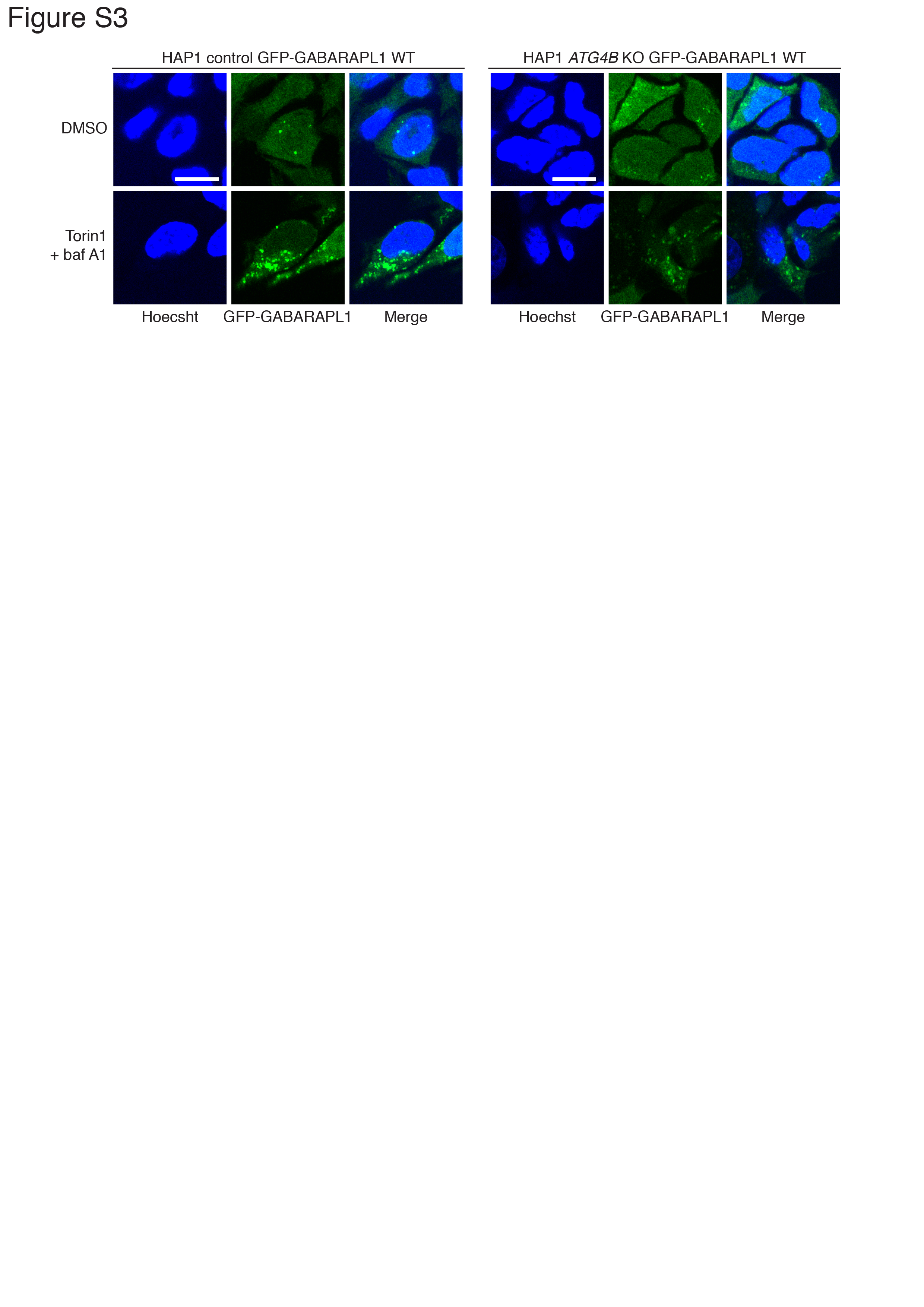


**Figure S3.** Stably expressed GFP-GABARAPL1 WT localizes to puncta in the absence of ATG4B. HAP1 control and *ATG4B* KO cells stably expressing GFP-GABARAPL1 WT were treated for 3 h with DMSO or 250 nM Torin1 + 10 nM baf A1 prior to fixation and confocal imaging. Scale bar: 10 µm.


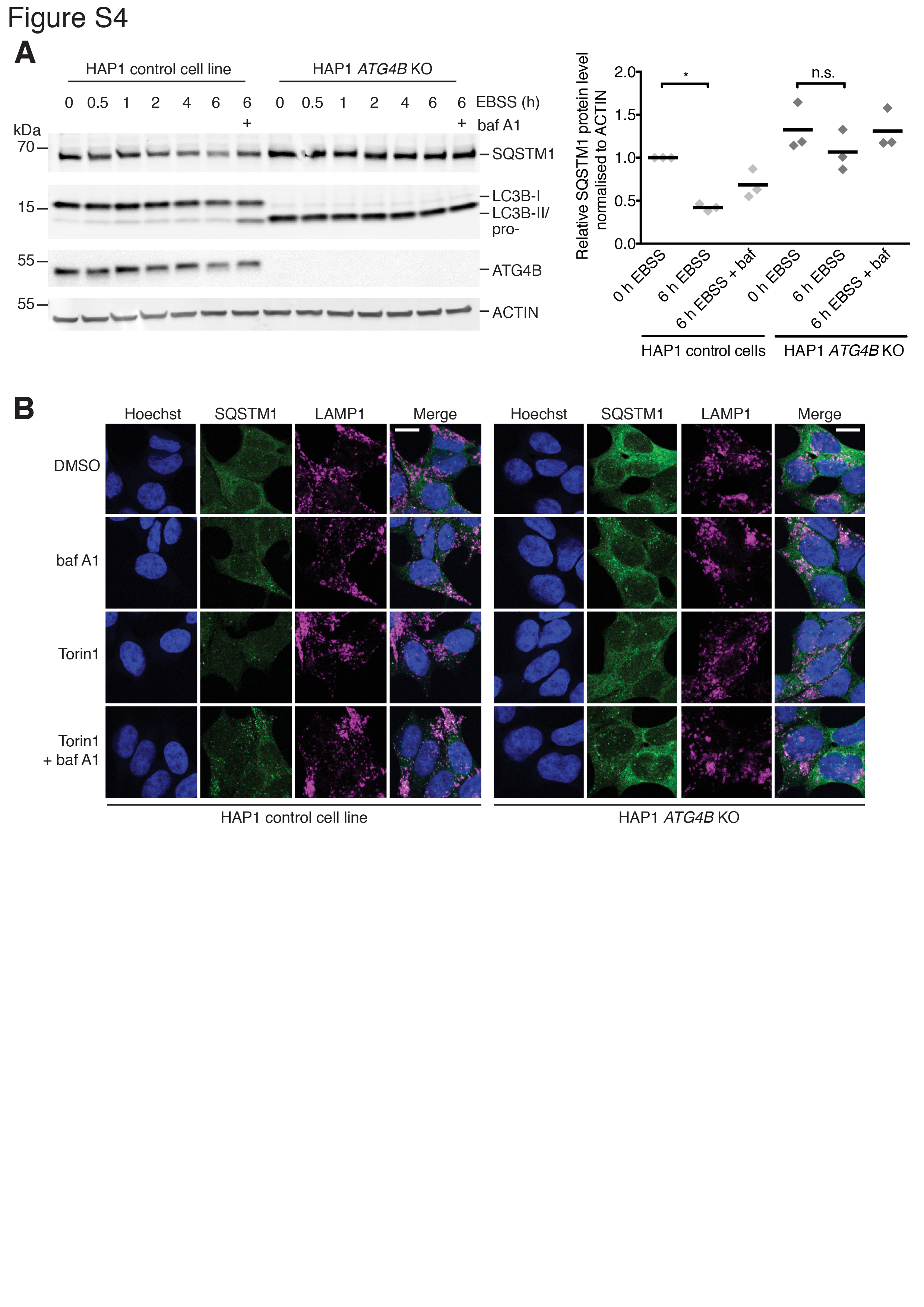


**Figure S4.** Defect in starvation and Torin1-induced SQSTM1 degradation in HAP1 cells lacking ATG4B. (**A**) Western blot of lysates (lacking NEM) from HAP1 control and *ATG4B* KO cells starved for incremental time points by washing twice with PBS and incubating with EBSS (or in the presence of 10 nM baf A1). Representative blots are shown on the left hand side. Protein level of SQSTM1 from 3 independent experiments was quantified by densitometry and plotted (right panel) after normalizing against the ACTIN protein level. Individual data points are shown and mean is displayed as a line. * P ≤ 0.05, n.s. P > 0.05 (Sidak’s multiple comparison test). (**B**) Immunocytochemistry of endogenous SQSTM1 and LAMP1 in control and *ATG4B* KO HAP1 cells. Cells were treated for 3 h with DMSO or 250 nM Torin1 + 10 nM baf A1 prior to fixation and staining. Scale bar: 10 µm.

**
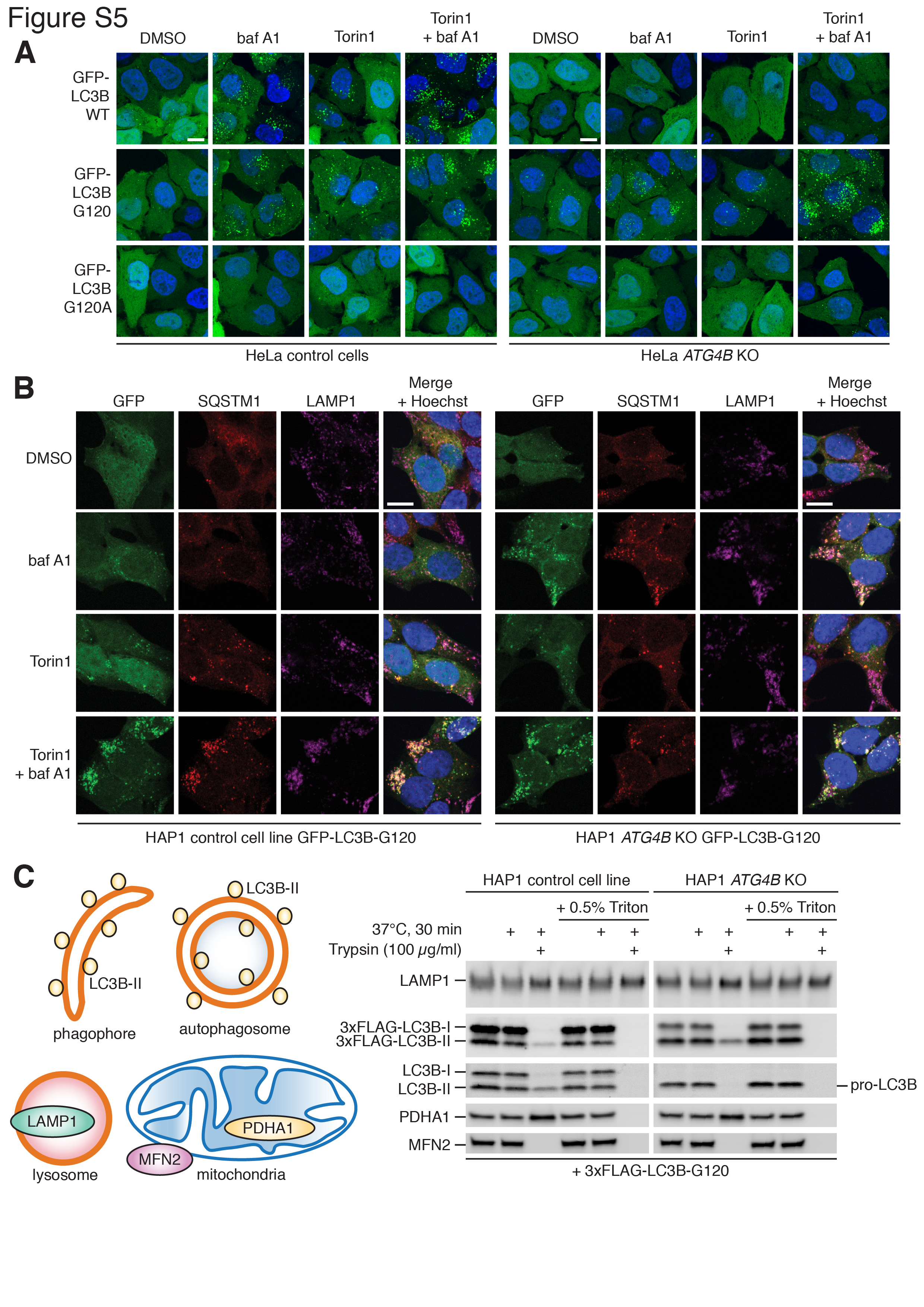
**

**Figure S5.** Additional characterization of primed LC3B expression in ATG4B deficient HAP1 and HeLa cells. (**A**) Confocal microscopy of GFP-tagged LC3B mutants stably expressed in HeLa control and *ATG4B* KO cells treated for 3 h with combinations of DMSO or 250 nM Torin1 with or without 10 nM baf A1. Scale bar: 10 µm. (**B**) Immunocytochemistry of endogenous SQSTM1 and LAMP1 in HAP1 control and *ATG4B* KO cells stably expressing GFP-LC3B-G120. Cells were treated for 3 h with combinations of DMSO or 250 nM Torin1 with or without 10 nM baf A1 for 3 h prior to fixation and staining. Scale bar: 10 µm. (**C**) Protease protection assay of homogenates from HAP1 control and *ATG4B* KO cells transfected with a plasmid encoding 3xFLAG-LC3B-G120 and treated for 3 h with 250 nM Torin1 and 10 nM baf A1. Diagrams (right hand side) show subcellular localization of target proteins detected by western blotting (left hand side).


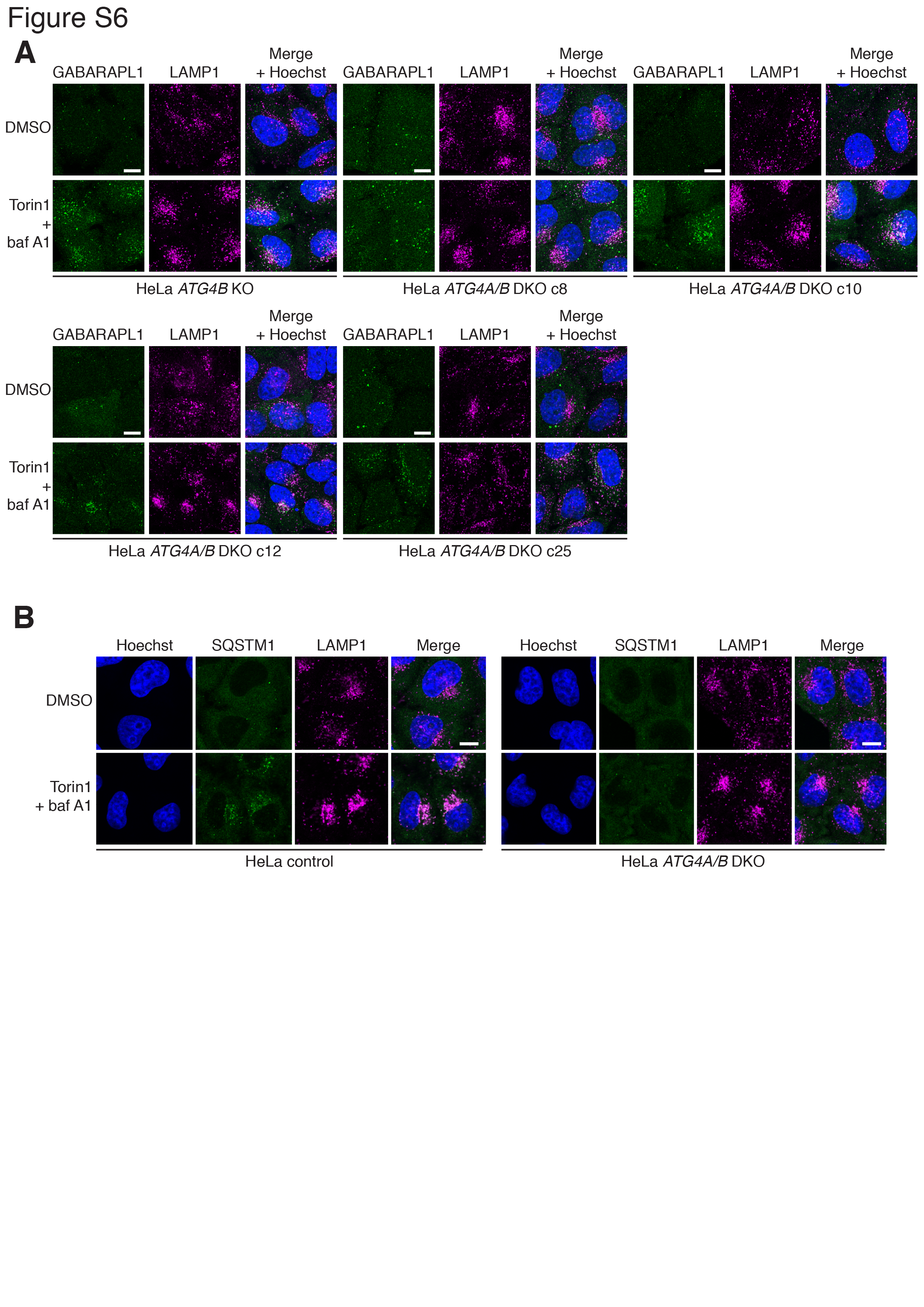


**Figure S6.** Additional characterization of HeLa *ATG4A/B* DKO cells. (**A**) Immunocytochemistry of endogenous GABARAPL1 and LAMP1 in HeLa *ATG4B* KO and different clones of *ATG4A/B* DKO cells treated for 3 h with DMSO or 250 nM Torin1 + 10 nM baf A1. Scale bar: 10 µm. (**B**) Immunocytochemistry of endogenous SQSTM1 and LAMP1 in HeLa control and *ATG4A/B* DKO (c25) cells treated with DMSO or 250 nM Torin1 + 10 nM baf A1 for 3 h. Scale bar: 10 µm.

**
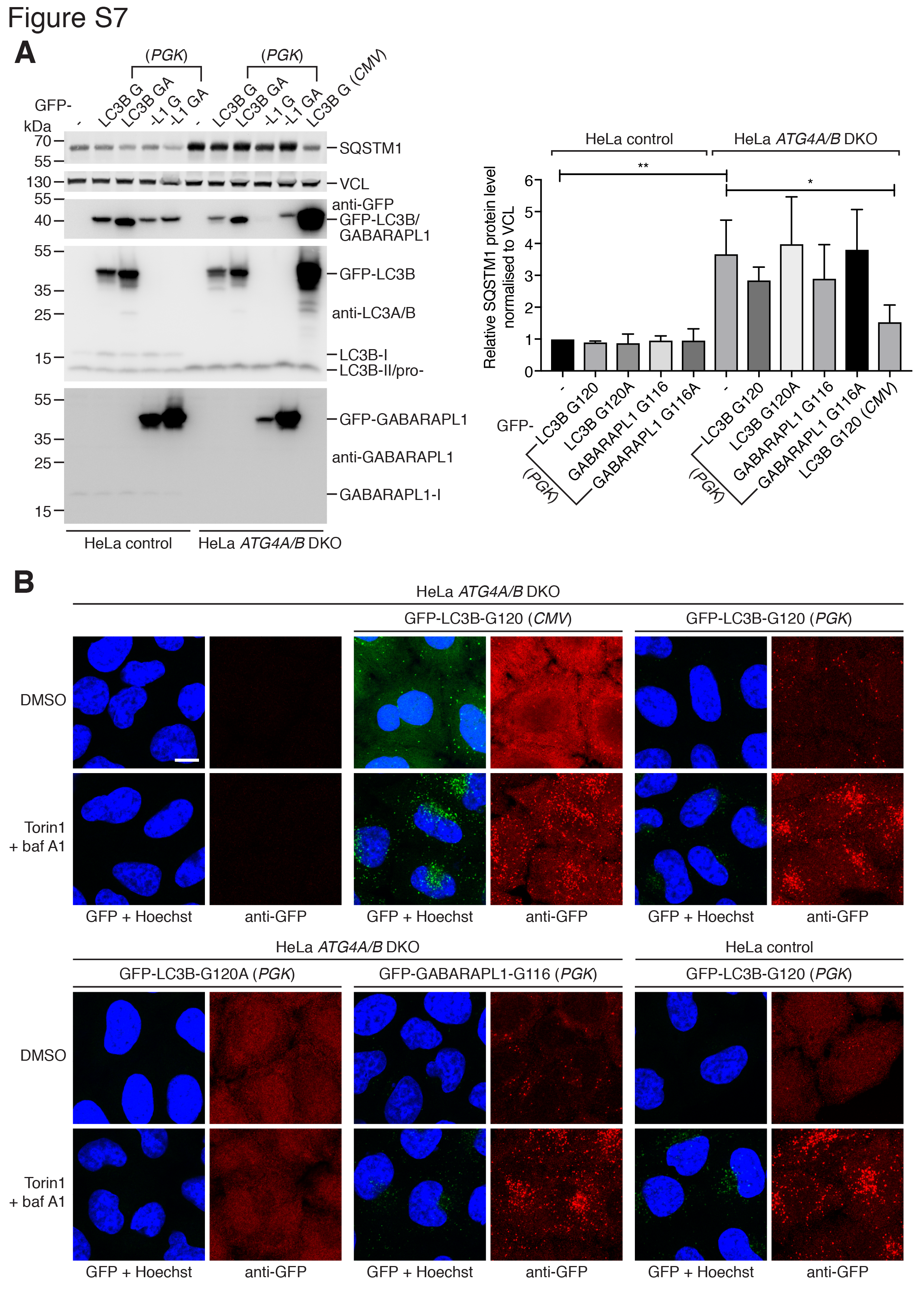
**

**Figure S7.** Validation of HeLa cells stably expressing reduced levels of *PGK* promoter-driven GFP-LC3B or GABARAPL1 bypass mutants. (**A**) Western blot analysis of HeLa control and *ATG4A/B* DKO (c25) cells, either untransduced (-) or stably expressing lentiviral-delivered GFP-LC3B-G120/-G120A (G/GA) or GFP-GABARAPL1-G116/-G116A (G/GA) under control of the low expression *PGK* promoter. As a positive control, *ATG4A/B* DKO (c25) cells stably expressing *CMV*-driven GFP-LC3B-G120 (as shown in Figures 7C-E) were assessed in parallel. Densitometry quantification of basal SQSTM1 protein level in each cell line from 3 independent experiments is shown on the right hand side. ** P ≤ 0.01, * P ≤ 0.05, differences between other groups were not significant (P > 0.05, Sidak’s multiple comparison test). (**B**) Immunocytochemistry of GFP localization in cells shown in Figure S7A treated with DMSO or 250 nM Torin1 + 10 nM baf A1 for 3 h. Native GFP fluorescence (shown in green) was difficult to detect at reduced expression levels. To reveal GFP construct localization, cells were stained using anti-GFP antibody that was detected on a separate channel shown in red. Scale bar: 10 µm.

**
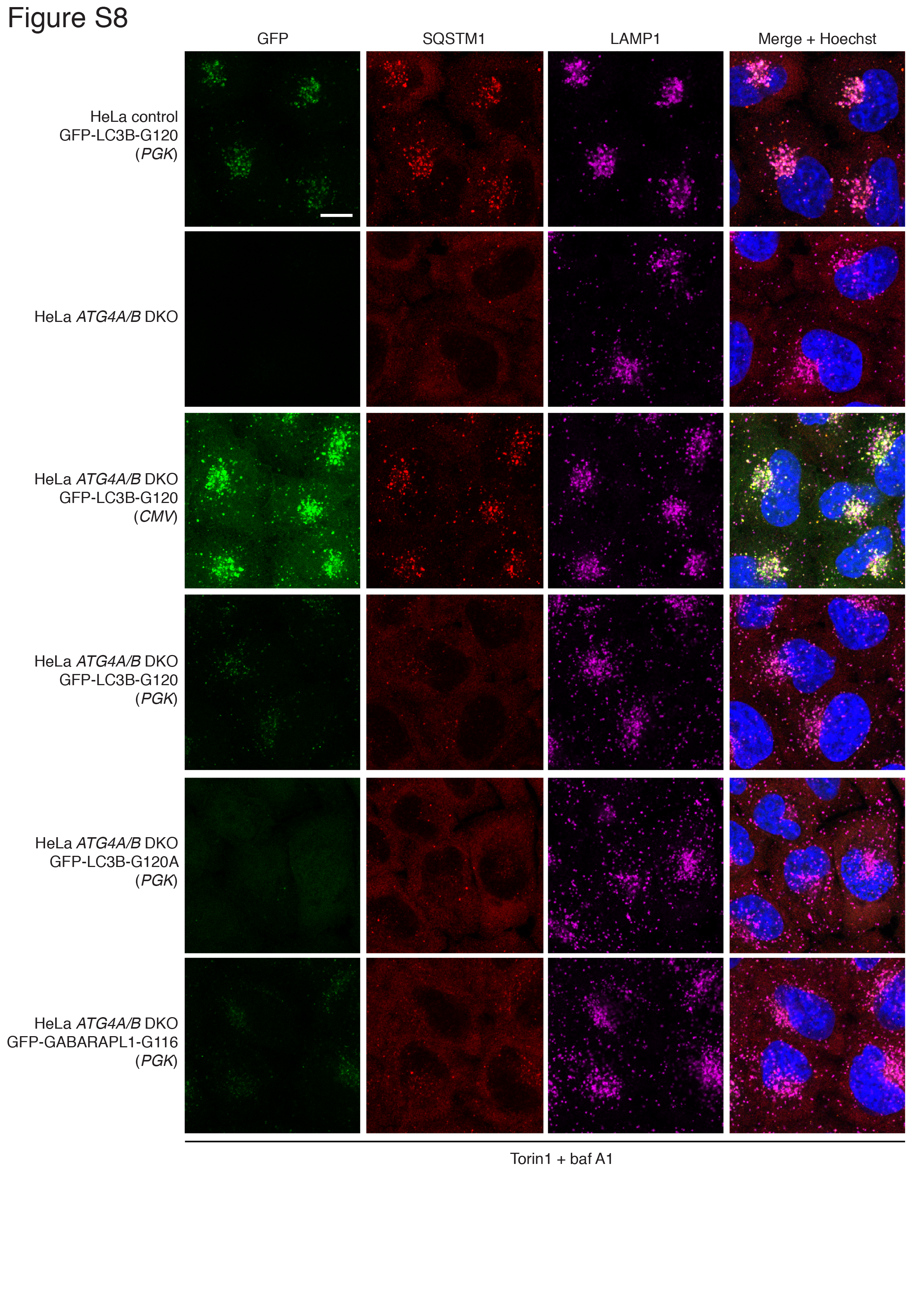
**

**Figure S8.** Reduced efficiency of SQSTM1 lysosome delivery in *ATG4A/B* DKO cells expressing low levels of GFP-tagged LC3B or GABARAPL1 bypass mutants compared to high expression of GFP-LC3B-G120. Immunocytochemistry of endogenous SQSTM1 and LAMP1 in cells shown in Figure S7A treated for 3 h with 250 nM Torin1 + 10 nM baf A1 prior to fixation and staining. Native GFP fluorescence is shown. Scale bar: 10 µm.

**
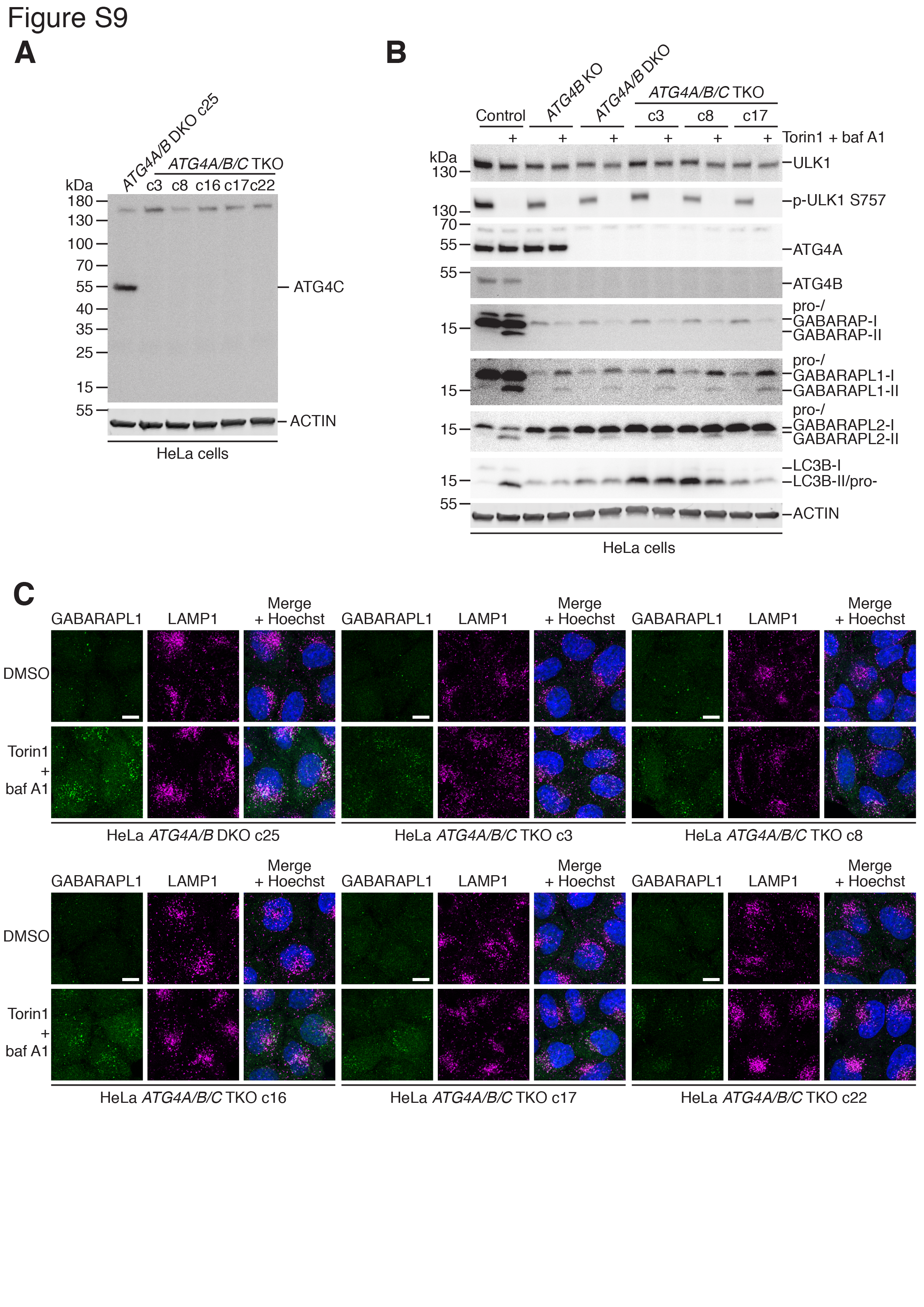
**

**Figure S9.** Additional characterization of HeLa *ATG4A/B/C* TKO cells. (**A**) Western blot analysis of HeLa *ATG4A/B* DKO (c25) and different clones of HeLa *ATG4A/B/C* TKO cells lysed without NEM and probed with anti-ATG4C. (**B**) Western blot analysis of HeLa control, *ATG4B* KO, *ATG4A/B* DKO (c25) cells and different *ATG4A/B/C* TKO clones treated for 3 h with DMSO or 250 nM Torin1 + 10 nM baf A1. (**C**) Immunocytochemistry of endogenous GABARAPL1 and LAMP1 in HeLa *ATG4A/B* DKO cells and different *ATG4A/B/C* TKO cell clones treated with DMSO or 250 nM Torin1 + 10 nM baf A1 for 3 h. Scale bar: 10 µm.


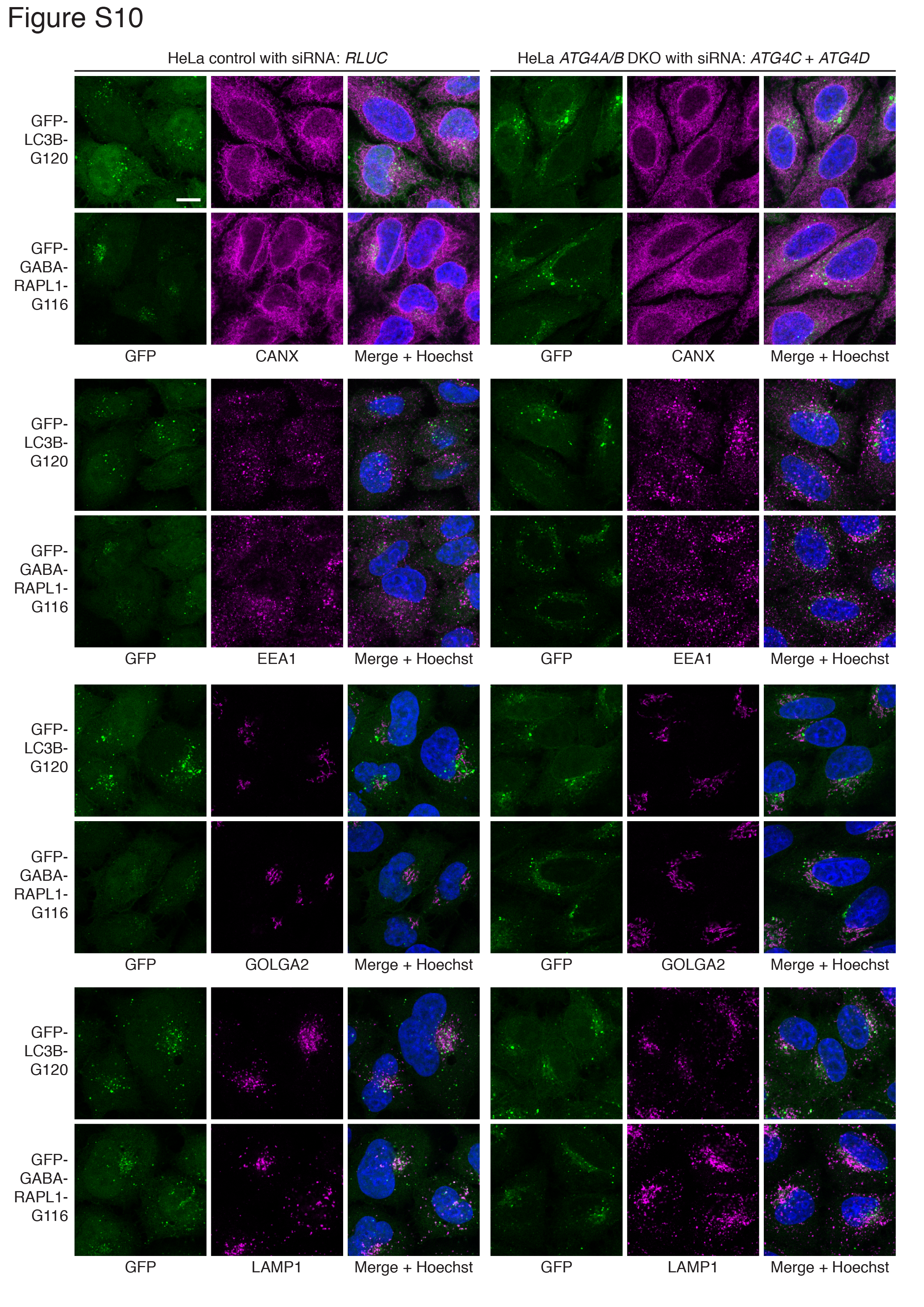


# Figure S10. Delipidation by ATG4 isoforms is not necessary to prevent mislocalization of LC3/GABARAP to non-autophagic organelles. HeLa control and *ATG4A/B* DKO (c25) cells stably expressing GFP-LC3B-G120 or GFP-GABARAPL1-G116 were transfected with siRNA targeting *RLUC* or *ATG4C* + *ATG4D* prior to immunocytochemistry and confocal microscopy to assess GFP localization. Cells were stained with antibodies targeting different organelle markers: CANX (calnexin; endoplasmic reticulum), EEA1 (early endosome antigen 1), GOLGA2 (golgin A2; Golgi) and LAMP1 (lysosomal associated membrane protein 1). Scale bar: 10 µm.

**Table S1.** Details of genomic loci and oligonucleotides used for CRISPR-Cas9 genome editing in HeLa cells, as well as PCR primers used for genomic validation by Sanger sequencing.

| **Gene targeted (exon targeted)** | **Target location (Reference assembly GRCh38)** | **CRISPR ID/(source)** | **19 nucleotide guide sequence** | **Guide oligo (+) (5' to 3')** | **Guide oligo (-) (5' to 3')** | **Forward genomic primer (5' to 3')** | **Reverse genomic primer (3' to 5')** |
| --- | --- | --- | --- | --- | --- | --- | --- |
| *ATG4A* (exon 2) | X:108126133-108126155 | ID: 1195746731 (Sanger CRISPR finder) | ATACCAGCTCATCTGTATC | CACCGATACCAGCTCATCTGTATC | AAACGATACAGATGAGCTGGTATC | GGGGACAAGTTTGTACAAAAAAGCAGGCTACAAAGAAGGCCCCATGGTA | GGGGACCACTTTGTACAAGAAAGCTGGGTTCTAGAATTTGGGGATTCTCCTTT |
| *ATG4B* (exon 4) | 2:241653584-241653606 | ID: 949006718  (Sanger CRISPR finder) | CCTAGGTGCCGGCACACCA | CACCGCCTAGGTGCCGGCACACCA | AAACTGGTGTGCCGGCACCTAGGC | GGGGACAAGTTTGTACAAAAAAGCAGGCTTAGTTACTGAGTCCTAAGAGGTGTGTGTTG | GGGGACCACTTTGTACAAGAAAGCTGGGTACATTACTCAAGAAACCCCCAAGTTC |
| *ATG4C* (exon 4) | 1:62816613-62816635 | ID: 908305971  (Sanger CRISPR finder) | TAGAGGATCACGTAATTGC | CACCGTAGAGGATCACGTAATTGC | AAACGCAATTACGTGATCCTCTAC | GGGGACAAGTTTGTACAAAAAAGCAGGCTAGGCAGTCAGGAAAATCTAACT | GGGGACCACTTTGTACAAGAAAGCTGGGTCTCAGGGAATTTCATGCAGTTTGT |
| Non-targeting | N/A | rg_0311  (GeCKO library v2) (Sanjana et al., 2014) | GGCCCGCATAGGATATCGC | CACCGGGCCCGCATAGGATATCGC | AAACGCGATATCCTATGCGGGCCC | N/A | N/A |

**Table S2.** Sequences of primers and DNA templates used to generate Gateway entry clones for LC3/GABARAP coding sequences.

| **Gene/entry clone** | **RefSeq of PCR template** | **Forward primer sequence**  **(5' to 3')** | **Reverse primer sequence**  **(5' to 3')** |
| --- | --- | --- | --- |
| *MAP1LC3A* | NM_032514.3 | GGGGACAAGTTTGTACAAAAAAGCAGGCTTAATGCCCTCAGACCGGCCTTTC | GGGGACCACTTTGTACAAGAAAGCTGGGTATTAGAAGCCGAAGGTTTCCTGGGAG |
| *MAP1LC3A* (no stop codon) | NM_032514.3 | GGGGACAAGTTTGTACAAAAAAGCAGGCTTAATGCCCTCAGACCGGCCTTTC | GGGGACCACTTTGTACAAGAAAGCTGGGTAGAAGCCGAAGGTTTCCTGG |
| *MAP1LC3A* (G120A) | NM_032514.3 | GGGGACAAGTTTGTACAAAAAAGCAGGCTTAATGCCCTCAGACCGGCCTTTC | GGGGACCACTTTGTACAAGAAAGCTGGGTATTAGAAGGCGAAGGTTTCCTGGGAG |
| *MAP1LC3A* (G120) | NM_032514.3 | GGGGACAAGTTTGTACAAAAAAGCAGGCTTAATGCCCTCAGACCGGCCTTTC | GGGGACCACTTTGTACAAGAAAGCTGGGTATTAGCCGAAGGTTTCCTGGGAGG |
| *MAP1LC3B* | NM_022818.4 | GGGGACAAGTTTGTACAAAAAAGCAGGCTTAATGCCGTCGGAGAAGACC | GGGGACCACTTTGTACAAGAAAGCTGGGTATTACACTGACAATTTCATCCCGAAC |
| *MAP1LC3B* (no stop codon) | NM_022818.4 | GGGGACAAGTTTGTACAAAAAAGCAGGCTTAATGCCGTCGGAGAAGACC | GGGGACCACTTTGTACAAGAAAGCTGGGTACACTGACAATTTCATCCCG |
| *MAP1LC3B* (G120A) | NM_022818.4 | GGGGACAAGTTTGTACAAAAAAGCAGGCTTAATGCCGTCGGAGAAGACC | GGGGACCACTTTGTACAAGAAAGCTGGGTATTACACTGACAATTTCATCGCGAACGTC |
| *MAP1LC3B* (G120A; no stop codon) | NM_022818.4 | GGGGACAAGTTTGTACAAAAAAGCAGGCTTAATGCCGTCGGAGAAGACC | GGGGACCACTTTGTACAAGAAAGCTGGGTACACTGACAATTTCATCGCG |
| *MAP1LC3B* (G120) | NM_022818.4 | GGGGACAAGTTTGTACAAAAAAGCAGGCTTAATGCCGTCGGAGAAGACC | GGGGACCACTTTGTACAAGAAAGCTGGGTATTACCCGAACGTCTCCTGGG |
| *MAP1LC3B2* (no stop codon) | NM_001085481.1 | GGGGACAAGTTTGTACAAAAAAGCAGGCTTAATGCCGTCGGAGAAGACC | GGGGACCACTTTGTACAAGAAAGCTGGGTACACTGACAATTTCATCCCG |
| *MAP1LC3C* | NM_001004343.2 | GGGGACAAGTTTGTACAAAAAAGCAGGCTTAATGCCGCCTCCACAGAAAATCCC | GGGGACCACTTTGTACAAGAAAGCTGGGTATTAGAGAGGATTGCAGGGTCTGTCC |
| *MAP1LC3C* (no stop codon) | NM_001004343.2 | GGGGACAAGTTTGTACAAAAAAGCAGGCTTAATGCCGCCTCCACAGAAAATCCC | GGGGACCACTTTGTACAAGAAAGCTGGGTAGAGAGGATTGCAGGGTCTG |
| *MAP1LC3C* (G126) | NM_001004343.2 | GGGGACAAGTTTGTACAAAAAAGCAGGCTTAATGCCGCCTCCACAGAAAATCCC | GGGGACCACTTTGTACAAGAAAGCTGGGTATTAGCCAAATGTCTCCTGGGAGGC |
| *GABARAP* | NM_007278.1 | GGGGACAAGTTTGTACAAAAAAGCAGGCTTAATGAAGTTCGTGTACAAAGAAGAG | GGGGACCACTTTGTACAAGAAAGCTGGGTATTACAGACCGTAGACACTTTCGTC |
| *GABARAP* (no stop codon) | NM_007278.1 | GGGGACAAGTTTGTACAAAAAAGCAGGCTTAATGAAGTTCGTGTACAAAGAAGAG | GGGGACCACTTTGTACAAGAAAGCTGGGTACAGACCGTAGACACTTTCG |
| *GABARAP* (G116A) | NM_007278.1 | GGGGACAAGTTTGTACAAAAAAGCAGGCTTAATGAAGTTCGTGTACAAAGAAGAG | GGGGACCACTTTGTACAAGAAAGCTGGGTATTACAGAGCGTAGACACTTTCGTC |
| *GABARAP* (G116) | NM_007278.1 | GGGGACAAGTTTGTACAAAAAAGCAGGCTTAATGAAGTTCGTGTACAAAGAAGAG | GGGGACCACTTTGTACAAGAAAGCTGGGTATTAACCGTAGACACTTTCGTCAC |
| *GABARAPL1* | NM_031412.2 | GGGGACAAGTTTGTACAAAAAAGCAGGCTTAATGAAGTTCCAGTACAAGGAGG | GGGGACCACTTTGTACAAGAAAGCTGGGTATTATTTCCCATAGACACTCTCATCAC |
| *GABARAPL1* (no stop codon) | NM_031412.2 | GGGGACAAGTTTGTACAAAAAAGCAGGCTTAATGAAGTTCCAGTACAAGGAGG | GGGGACCACTTTGTACAAGAAAGCTGGGTATTTCCCATAGACACTCTCATC |
| *GABARAPL1* (G116A) | NM_031412.2 | GGGGACAAGTTTGTACAAAAAAGCAGGCTTAATGAAGTTCCAGTACAAGGAGG | GGGGACCACTTTGTACAAGAAAGCTGGGTATTATTTCGCATAGACACTCTCATCAC |
| *GABARAPL1* (G116) | NM_031412.2 | GGGGACAAGTTTGTACAAAAAAGCAGGCTTAATGAAGTTCCAGTACAAGGAGG | GGGGACCACTTTGTACAAGAAAGCTGGGTATTACCCATAGACACTCTCATCACTG |
| *GABARAPL2* | NM_007285.6 | GGGGACAAGTTTGTACAAAAAAGCAGGCTTAATGAAGTGGATGTTCAAGGAGG | GGGGACCACTTTGTACAAGAAAGCTGGGTATTAGAAGCCAAAAGTGTTCTCTCC |
| *GABARAPL2* (no stop codon) | NM_007285.6 | GGGGACAAGTTTGTACAAAAAAGCAGGCTTAATGAAGTGGATGTTCAAGGAGG | GGGGACCACTTTGTACAAGAAAGCTGGGTAGAAGCCAAAAGTGTTCTCTC |
| GABARAPL2 G116A | NM_007285.6 | GGGGACAAGTTTGTACAAAAAAGCAGGCTTAATGAAGTGGATGTTCAAGGAGG | GGGGACCACTTTGTACAAGAAAGCTGGGTATTAGAAGGCAAAAGTGTTCTCTCC |
| *GABARAPL2* (G116) | NM_007285.6 | GGGGACAAGTTTGTACAAAAAAGCAGGCTTAATGAAGTGGATGTTCAAGGAGG | GGGGACCACTTTGTACAAGAAAGCTGGGTATTAGCCAAAAGTGTTCTCTCCGC |

**Table S3.** Mutations detected by Sanger sequencing of PCR-amplified genomic DNA in main CRISPR KO HeLa clones used in this study.

| **Cell name** | **Main clone** | **Parental cell** | **Gene targeted using CRISPR** | **Mutations detected in targeted gene (no. of Sanger sequence reads detected/total)** |
| --- | --- | --- | --- | --- |
| HeLa *ATG4B* KO | N/A | HeLa | *ATG4B* | NM_013325.4:c.262_264del (13/19)  NM_013325.4:c.263_264insCT (6/19) |
| HeLa *ATG4A/B* DKO | c25 | HeLa *ATG4B* KO | *ATG4A* | NM_052936.4:c.73_83del (5/5) |
| HeLa *ATG4A/B/C* TKO | c22 | HeLa *ATG4A/B* DKO c25 | *ATG4C* | NM_032852.3:c.216delT;223A>C (1/4)  NM_032852.3:c.181_247del (1/4)  NM_032852.3:c.216_217insT (1/4)  NM_032852.3:c.213_215del (1/4) |
